# EPI-DOM framework for assessing fish welfare: external and internal indicators for tilapia

**DOI:** 10.3389/fvets.2026.1770985

**Published:** 2026-04-13

**Authors:** Rosario Martínez-Yáñez

**Affiliations:** Aquaculture Laboratory, Departamento de Veterinaria y Zootecnia, División de Ciencias de la Vida, Universidad de Guanajuato, Irapuato, Guanajuato, Mexico

**Keywords:** animal welfare, welfare assessment, EPI-DOM, aquaculture, tilapia, indicators, risk factors, management

## Abstract

**Introduction:**

The EPI-DOM framework proposes an applied epidemiological approach to evaluate and manage tilapia welfare in laboratory and production systems, integrating animal-based indicators with risk factors organized into operational domains (management, environment, and interaction).

**Methods:**

An integrative review (2000–2025) was conducted and analytically structured under the EPI-DOM framework to: (i) classify external and internal indicators and define adverse welfare events (AWE); (ii) organize risk factors within the Management domain; and (iii) link indicators to risk factors through cross-mapping to prioritize interventions. Methodological criteria were incorporated for contextual interpretation of body integrity, physiological and biochemical ranges, harmonization of units/methods (for farms and laboratories), and the design of practical sampling schemes (population-level and sentinel) for farms.

**Results:**

The final product is an operational guide that translates dispersed evidence into a replicable welfare assessment system, including domain-based risk matrices, checklists, and preventive/corrective action guidance to identify critical welfare points and support context-specific decision-making.

**Discussion:**

EPI-DOM bridges welfare science and applied epidemiology by preserving traceability (indicator–risk–management–intervention), promoting comparability across systems, and allowing operational thresholds and sampling strategies to be adapted to local conditions without compromising methodological consistency.

**Conclusions:**

This EPI-DOM–aligned guide provides a dynamic, verifiable framework to support welfare improvement in tilapia, enabling evidence-based decision-making and implementation of good practices in both laboratory and field settings.

## 1 Introduction

### 1.1 Global context of animal welfare in aquaculture: advances, challenges, and the need for integrative frameworks

Animal welfare in aquaculture is a complex, multidisciplinary field in which breeding methods (1, 2), feeding practices (3, 4), environmental conditions (2, 5), technological innovations (6), and regulatory frameworks (7, 8) interact dynamically to shape fish health and performance outcomes. Breeding and rearing strategies influence disease resistance, stress, and species-specific behavioral needs (1, 2), while feeding strategies—including functional feeds and alternative formulations—can support immunity, health, and welfare, although extrapolation across contexts remains limited by short study durations and strong species-specific focus (9–11). Balancing nutritional adequacy with environmental sustainability remains a central challenge (12, 13). The rearing environment shapes physiological and behavioral resilience; improving water quality, managing stocking density, and providing enrichment are associated with reduced stress and improved welfare (2, 5, 14). Controlled systems (e.g., biofloc, RAS, IMTA) may enhance welfare while supporting sustainability by mitigating pollution and optimizing resource use (6, 15, 16). Technological innovations enable non-invasive, real-time monitoring (e.g., computer vision, digital morphometry, wearable biosensors) (244), yet adoption remains constrained by environmental variability, species diversity, and particularly limited indicator standardization (2, 17–20). Adoption is also heterogeneous and often depends on company size, operational scale, and profit margins, which shape investment capacity, infrastructure, and access to technical expertise. From an ethical and regulatory perspective, recognition of fish sentience has strengthened the moral obligation to safeguard welfare, yet gaps persist in specificity and enforcement, and stakeholder perceptions remain heterogeneous, supporting the need for more holistic policy approaches (7, 21–24). Here, “policy approaches” refers broadly to governance instruments relevant to aquaculture welfare (e.g., public regulations, certification schemes, industry standards, and farm-level audit protocols) (242), and “ethical values” refers to welfare principles underpinning these instruments, including recognition of fish sentience, prevention of avoidable suffering, minimization of pain and distress, and—where feasible—the promotion of positive welfare states. In practice, these gaps often translate into limited species- and system-specific operational guidance on which animal-based indicators to prioritize, how to define thresholds/severity scales, and how to translate findings into auditable corrective actions; EPI-DOM addresses these voids by linking indicators to modifiable risk factors within domains and structuring decision outputs (sentinel triggers, risk prioritization, and SOP-based actions). Finally, key knowledge gaps remain regarding animal-based indicators, species-specific metrics, validation of non-lethal biomarkers, and standardized welfare protocols (4, 23, 25–27).

### 1.2 Evolution of animal welfare frameworks: from the Five Freedoms to the Five Domains

The historical evolution of animal welfare frameworks reflects a progressive expansion in concept and methodology that has reshaped how welfare is understood and assessed across species (20). The Five Freedoms, formulated in the mid-20th century, established a foundational set of ethical and practical principles centered on preventing suffering—freedom from hunger and thirst; from discomfort; from pain, injury, or disease; from fear and distress; and to express normal behavior (28, 29). While widely adopted in education, assessment, and legislation, this framework has been criticized for its idealized and predominantly negative orientation, emphasizing the absence of suffering more than the presence of positive welfare states (20). Welfare science subsequently shifted toward more comprehensive models such as the Five Domains, which incorporate both physical and mental dimensions and explicitly include positive experiences, aligning with growing recognition of animal sentience and related ethical and legislative developments (30–34, 245). The inclusion of behavioral interactions and the human–animal relationship further broadened evaluation by emphasizing control, choice, and engagement as drivers of positive affective states (35–37, 243). Increasingly, welfare is understood as an outcome contingent on human decisions that define and transform living conditions, where risk factors directly or indirectly determine physical and mental states, making their monitoring and control an ethical and operational responsibility (20). Nevertheless, operational challenges persist objective measurement of physical state and inference of mental states remain complex, requiring multi-indicator interpretation, and cultural and ethical differences continue to shape application across contexts, underscoring the need for adaptive, region- and species-sensitive approaches (38, 39). According to the Terrestrial Code of the World Organization for Animal Health (OMSA, formerly OIE), animal welfare is defined as “the physical and mental state of an animal in relation to the conditions in which it lives and dies” (40), which provides an operational basis to integrate animal-based indicators with the conditions and decisions that generate them. In practice, however, the way welfare is defined, prioritized, and enforced varies across major aquaculture-producing regions. For example, Norway has an established legal framework for animal welfare that explicitly applies to animals kept by humans and is complemented by sector-specific aquaculture regulations requiring that operations ensure health and welfare standards for fish (41). In contrast, in China—currently the world's largest fish producer—fish are commonly transported live across the supply chain and published evidence indicates heterogeneous stakeholder awareness and limited sector-wide welfare standardization in practice, underscoring the need for tools that translate welfare principles into measurable indicators and auditable actions under diverse production realities (42).

### 1.3 EPI-DOM framework: integrating epidemiology and emerging welfare domains

The EPI-DOM approach (EPIdemiology-DOMains) was proposed by Martínez-Yáñez et al. (20) as a conceptual and methodological framework for comprehensive assessment of integral animal welfare, addressing limitations of classical welfare models. EPI-DOM integrates applied veterinary epidemiology with a welfare-domain structure, enabling welfare to be interpreted not only as an observable state but as a dynamic, management-driven process shaped by quantifiable risk factors that can be modified through human decision-making. In this context, a “dynamic management-driven process” means that welfare is monitored over time through repeated indicator measurement and surveillance of risk factors and is actively managed through preventive and corrective actions that adjust system conditions to reduce the probability of adverse welfare events and promote positive states. This manuscript builds on the foundational EPI-DOM framework by providing a species-specific operational application for tilapia, translating the model into a practical guide that integrates external and internal indicators with interpretive criteria (AWE, sentinel triggers, and population-based thresholds) and decision-oriented outputs for preventive and corrective actions (20).

#### 1.3.1 Need for a new integrative framework

Traditional frameworks (e.g., Five Freedoms and Five Domains) have advanced welfare thinking, yet their application is limited when observable indicators are not explicitly connected to the underlying causes that generate them (28, 29, 31, 32). Even where welfare assessment methods are available, practical uptake and comparability are constrained when indicators lack standardized interpretation and do not translate into actionable decision outputs for farms (43). In aquaculture, “risk” is also commonly framed at the industry and governance level (e.g., operational, market, regulatory, and financial risk sources), highlighting that management decisions are shaped by multi-layered constraints beyond biology; however, these approaches do not operationalize welfare as epidemiological adverse events linked to animal-based indicators (44). Recent work has further emphasized that welfare risks are unevenly distributed across aquatic taxa at the species level, reinforcing the need for species-relevant and operationalizable assessment tools rather than one-size-fits-all approaches (45). EPI-DOM proposes a causal link between welfare indicators and risk factors to support explanatory, preventive, and comparative analyses across production units and within a given system. Under this logic, welfare assessment becomes an adaptive, unit-specific (“tailor-made”) process based on operational context and real data, supporting early action before adverse welfare events become evident. EPI-DOM provides the structural backbone for identifying risk factors, estimating their likelihood, and designing preventive and corrective activities; the Management domain acts as the theoretical and normative axis, while Management–Environment and Management–Interaction integration translates theory into practical, unit-specific operational manuals (20).

#### 1.3.2 Epidemiological basis of the model

EPI-DOM adapts core principles of veterinary epidemiology—risk, risk factors, adverse events, and probability of occurrence—to establish causal relationships among system conditions, management practices, and animals' physiological and behavioral responses. Welfare is interpreted as the outcome of cumulative exposure to risk factors rather than as a fixed state, enabling identification of critical determinants, estimation of adverse event probability, and design of mitigation strategies. In this sense, EPI-DOM frames welfare as an observable, explanatory, and manageable process measured and improved through verifiable actions (20).

#### 1.3.3 Structural differentiation and analytical logic of EPI-DOM

**Figure 1:**
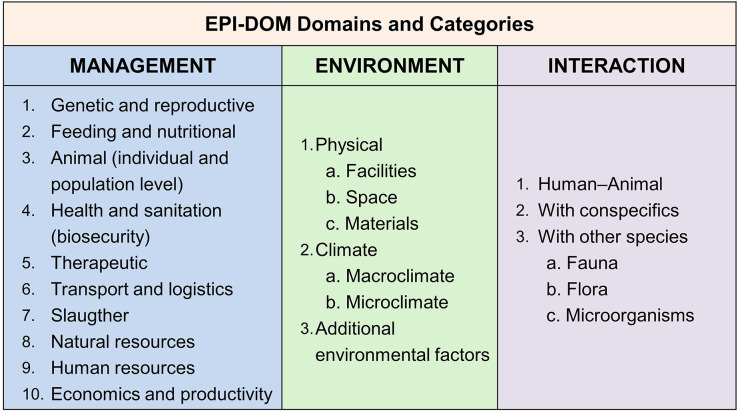
EPI-DOM domains (DOM) and categories used to organize welfare-related risk factors. The framework structures determinants of welfare into three domains—Management, Environment, and Interaction—each comprising operational categories that are evaluated relationally to identify critical risk points and guide preventive/corrective actions. Adapted from Martínez-Yáñez et al. (20).

EPI-DOM distinguishes three complementary analytical levels: (i) welfare indicators (animal-based indicators) measured directly in the animal and grouped as external, internal, and behavioral indicators, interpreted as adverse welfare events when they reflect functional compromise; (ii) risk factors (EPI), defined as conditions, practices, or system characteristics that increase the likelihood of adverse welfare events and may act cumulatively, interactively, or synergistically; and (iii) domains (DOM)—Management, Environment, and Interaction—within which risk factors are organized into categories and variables that influence welfare directly or indirectly. Analyses are relational, hierarchical, and multidimensional: management is cross evaluated against Environment to identify critical risk points, then integrated with Interaction to detect combined effects that may not be evident in one-dimensional assessments. This relational logic aligns with interaction-based causal structures proposed for complex adverse outcomes in aquaculture systems, where multiple environmental, biological, nutritional, and management drivers jointly shape event probability and require integrated management responses (46). This process supports identification and prioritization of adverse welfare events, definition of operational thresholds, and specification of evidence-based preventive and corrective actions. These actions are translated into preventive and corrective operational manuals that can be adapted to both laboratory and field conditions, supporting mitigation of negative welfare and promotion of positive welfare (20). The model's conceptual structure is schematically summarized in Figure 1 [modified from Martínez-Yáñez et al. (20)].

### 1.4 Relevance of tilapia (*Oreochromis* spp.)

Research on tilapia (*Oreochromis spp*.) is critical due to its major role in global aquaculture, food security, and experimental research. Since the 1970s, tilapia farming has expanded rapidly, becoming the world's second most widely cultivated finfish group, with annual production exceeding 6 million metric tons, driven largely by Nile tilapia (*Oreochromis niloticus*) because of its adaptability and growth performance (47, 48). Tilapia is widely farmed in tropical and subtropical regions, contributing substantially to protein supply and rural livelihoods, and its production has evolved through advances in selective breeding, nutrition, and diversified systems (e.g., semi-intensive ponds, RAS, and IMTA) (49–53). Its relevance is further supported by economic impact, environmental plasticity, and versatility as both a food source and a model organism (54, 55). *Oreochromis spp*. are also widely used as experimental models in genetic improvement, nutrition trials, disease-resistance assessments, and environmental stress research, highlighting their value for biomedical and ecological investigations (56–60). Given this biological, economic, and experimental relevance, tilapia provide an appropriate model species for application of the EPI-DOM framework, because their broad use across systems and measurable responses to risk factors support the definition and validation of species-relevant welfare indicators and the development of operational manuals tailored to tropical conditions. Within this context, the aim of the present article is to develop a practical guide for welfare assessment and management in tilapia (*Oreochromis* spp.) in aquaculture systems based on the EPI-DOM framework (Epidemiology + Domains). By integrating external and internal welfare indicators with risk factors associated with the Management domain, we propose an operational approach to identify adverse welfare events, interpret severity, and guide evidence-based preventive and corrective actions for both laboratory and production settings, strengthening prevention, continuous improvement, and ethical welfare management in aquaculture.

## 2 Materials and methods

### 2.1 Study design and methodological approach

This manuscript is an integrative narrative review with an analytical and applied orientation, designed to (i) interpret external and internal welfare indicators in tilapia from physiological and epidemiological perspectives and (ii) translate the evidence into a practical welfare assessment guide for laboratory, production, and field contexts. Rather than a purely descriptive synthesis, the review applies a structured process of classification and conceptual integration using EPI-DOM (EPIdemiology-DOMains) to link animal-based indicators to modifiable risk factors (EPI) and to the operational domains where corrective action is implemented (DOM).

### 2.2 Evidence identification, sources, and access

The review covered 2000–2025 and included peer-reviewed research articles, reviews, book chapters, and selected technical/regulatory documents relevant to fish welfare and aquaculture. Searches were performed in Scopus, Web of Science, PubMed, ScienceDirect, and Google Scholar (for complementary retrieval and technical documents). Authoritative normative and conceptual documents from internationally recognized bodies (e.g., WOAH, EFSA) were consulted when directly relevant to definitions and operational context. To strengthen transparency, evidence identification followed a protocol-inspired workflow: database searching with structured keyword combinations; title/abstract screening; full-text screening for methodological clarity and extractable information; backward/forward citation chaining from key articles; and structured extraction/synthesis under the EPI-DOM logic. Full texts were retrieved through institutional subscriptions and open-access sources when available; inaccessible full texts were not used to extract numerical thresholds or methodological details and were retained only when essential for conceptual context.

### 2.3 Search terms and eligibility criteria

Search strings were adapted to each database and iteratively refined. Core terms include: *Oreochromis niloticus*, tilapia, *Oreochromis spp*., fish welfare, aquatic animal welfare, physiological stress (acute/chronic), hematology, blood biochemistry, metabolic indicators, external indicators, respiratory/ventilation rate, heart rate, hypoxia, ammonia toxicity, water quality, handling stress, stocking density, management practices, risk factors, laboratory/field assessment, among others. Studies were included if they provided tilapia-relevant evidence on external indicators (body/tegument integrity) and/or internal physiological indicators (hematology, biochemistry, metabolism, respiration, and heart rate), including responses to environmental, handling, health-related, nutritional, or production stressors, even when “welfare” was not the primary stated objective. Exclusions comprised sources without verifiable scientific support, studies lacking clear methods, redundant publications, and studies where indicators could not be linked to welfare/stress/management interpretation.

### 2.4 Data extraction, synthesis, and indicator construction

Eligible sources were critically assessed and key information was extracted: indicator type (external/internal), direction of change, associated risk factor(s) (EPI), implicated operational domain/category (DOM), application context (laboratory/field/production), and practical relevance. Evidence was reorganized and integrated to identify consistent directional patterns while avoiding extrapolation of non-comparable absolute values across heterogeneous methods and contexts. The practical guide was constructed by organizing each indicator entry into: an operational description, physiological interpretation, linked risk factor(s) (EPI), implicated domain/category for action (DOM), applicability to laboratory/field settings, and corresponding corrective actions.

### 2.5 EPI-DOM framework used for interpretation

Indicator interpretation followed the EPI-DOM framework described by Martínez-Yáñez et al. (20). In brief, EPI denotes modifiable determinants (environmental, health-related, management, nutritional, among others) that increase the probability of welfare deterioration or adverse events, whereas DOM denotes the operational domains/management categories where failures materialize and where preventive/corrective measures are implemented (e.g., reproduction/genetics, feeding and nutrition, animal-related factors, health/sanitary management, therapeutics, transport/logistics, slaughter, water/natural resources, human resources, and economic/productive management). This structure distinguishes what is measured in the animal (indicators) from what causes or modulates it (risk factors), enabling a relational, decision-oriented synthesis.

### 2.6 Interpretive considerations

Compiled ranges reflect variability across studies (species/strain, size/age, temperature, salinity, density, system type, and analytical methods). Values should be interpreted within system-specific baselines; quantitative thresholds were extracted only when explicitly reported. Because methods differ across studies, methodological consistency is emphasized for longitudinal comparisons. The guide is intended as a decision-support tool for researchers and practitioners and does not replace professional judgment or system-tailored protocols.

## 3 Results

### 3.1 Conceptual foundation: the EPI-DOM framework (Epidemiology + Domains)

The EPI-DOM framework (EPIdemiology-DOMains) integrates veterinary epidemiology and animal welfare science to evaluate welfare as a dynamic, verifiable, and context-dependent process (20). In contrast to approaches that offer limited causal interpretability between management drivers and observed welfare states, EPI-DOM embeds epidemiological reasoning within a domain-based structure to connect animal responses to their determinants. Within this logic, adverse welfare events are treated as epidemiological events: determinants are identified, probability is interpreted in context, and preventive/corrective actions are defined, enabling objective welfare quantification through observable indicators linked to risk factors. EPI-DOM is grounded in three operational principles: (i) causality (linking conditions to animal responses), (ii) continuous surveillance (systematic monitoring of indicators and risk factors), and (iii) preventive and corrective action (translating assessment into decisions that reduce risk and promote positive welfare states).

### 3.2 Operational integration: from domains to action

EPI-DOM translates assessment into action through a relational, multidimensional analysis in which domain categories are cross-evaluated to identify critical risk points and define preventive and corrective measures. The Management domain comprises decision-driven risk factors (e.g., handling, nutrition, health management, biosecurity, genetics, personnel, economics); the Environment domain captures physicochemical conditions, infrastructure, and comfort; and the Interaction domain includes social relationships and human–animal interactions that shape behavior and mental state. This integration yields unit-specific animal welfare operational manuals for prevention, mitigation, and correction of identified risks, supporting continuous management toward more sustainable and ethically responsible production systems.

### 3.3 Aquaculture-specific adaptation: tilapia production cycle and critical control points (CCP)

**Figure 2:**
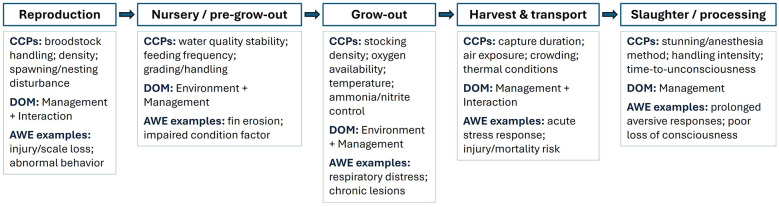
Tilapia production cycle and critical control points (CCPs) for welfare management within the EPI-DOM framework. Major production phases are shown together with typical CCPs, dominant DOM categories, and representative AWE examples to support rapid welfare-risk prioritization and operational decision-making.

Applied to tilapia (*Oreochromis* spp.) farming, EPI-DOM enables identification of critical control points across the production cycle—reproduction, nursery/pre-grow-out, grow-out, harvest/transport, and slaughter/processing—where welfare may be compromised. In each phase, adverse welfare events can be detected through animal-based indicators and interpreted within the context of domain-specific risk factors, particularly within Management as the decision-making axis. This approach links observations to practical, unit-specific protocols that support prevention, continuous welfare improvement, and development of species-relevant indicators and assessment scales for tilapia. Figure 2 summarizes the production cycle, highlights typical CCPs, links each CCP to the dominant EPI-DOM domain(s), and provides representative adverse welfare event (AWE) examples to support rapid risk prioritization and operational decision-making.

### 3.4 Practical guide for tilapia: operational definitions and decision rules

This section operationalizes EPI-DOM for farmed tilapia by focusing on external and internal indicators integrated into a scoring system consistent with applied epidemiological principles. The guide supports definition of risk levels, operational thresholds, and objective criteria for interpreting clinical and production-related findings, and it links each deviation to decision-oriented outputs (risk interpretation and corrective action). Behavioral indicators are introduced only to preserve conceptual integration; their full methodological development (subcategories, species-specific scales, and advanced analyses) will be presented in a subsequent contribution dedicated to behavioral assessment in tilapia.

#### 3.4.1 Standard Operating Procedure (SOPs) as the implementation unit

A Standard Operating Procedure (SOP) is a technical document that describes, in a sequential and verifiable manner, how a critical activity must be performed within a production or research system (61, 62). Within welfare programs, SOPs are essential because they standardize procedures across operators, reduce operational variability and human error, and make actions reproducible and auditable, linking daily practices to measurable welfare indicators (63–65). Within EPI-DOM, SOPs are the tool that translates domain findings into implementable and traceable actions (20). At minimum, a welfare-oriented SOP should specify: objective; protected indicator(s) (including severity and sentinel criteria when applicable); scope and linked domain(s); responsible personnel; required materials/equipment; stepwise procedure with critical control points and corrective actions; mandatory records; and review/update frequency.

#### 3.4.2 Adverse welfare event (AWE) and surveillance logic

An adverse welfare event (AWE) is defined as the simultaneous occurrence or repeated detection of indicators scoring ≥2 that are associated with a risk factor (EPI) or domain (DOM). Identifying an AWE directs mitigation toward the most likely root cause (e.g., biosecurity failure, suboptimal environmental parameters, inappropriate stocking density), strengthening traceability of preventive and corrective actions. Welfare assessment is recommended monthly or per batch, with intensified monitoring during high-risk events (e.g., grading, transport, vaccination, pre-harvest). Results should be recorded in a monitoring dashboard to detect trends, prioritize risks, and support continuous welfare improvement.

#### 3.4.3 Severity scoring and operational criteria (population-based and sentinel)

Each external and internal indicator is evaluated using a 0–2 severity scale designed to capture event intensity and its potential welfare impact. The 0–2 severity scoring was designed as a signal decision aid to promote timely, consistent interventions: 0 (green) indicates routine monitoring, 1 (yellow) indicates early warning requiring action within 24–48 h, and 2 (red) indicates a severe condition requiring immediate intervention and follow-up (Table 1).

**Table 1:** Scoring and operational criteria.

Score	Description	Operational interpretation	Signal
**0**	Normal/optimal state	**Routine detection**. Scheduled **preventive intervention** to minimize the future probability of an AWE (minor adjustments in management/environment/ interaction).	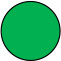
**1**	Mild to moderate alteration	**Early warning signs or AWE present without vital compromise. Requires corrective intervention within 24–48 h**, targeting the root cause identified within the model domains.	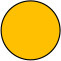
**2**	Severe alteration	**Consolidated AWE** with risk of significant pain, suffering, or mortality. Requires **immediate intervention** and continuous follow-up.	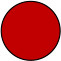

Bold values indicate the operational thresholds used for welfare interpretation and score assignment in this guide.

**Figure 3:**
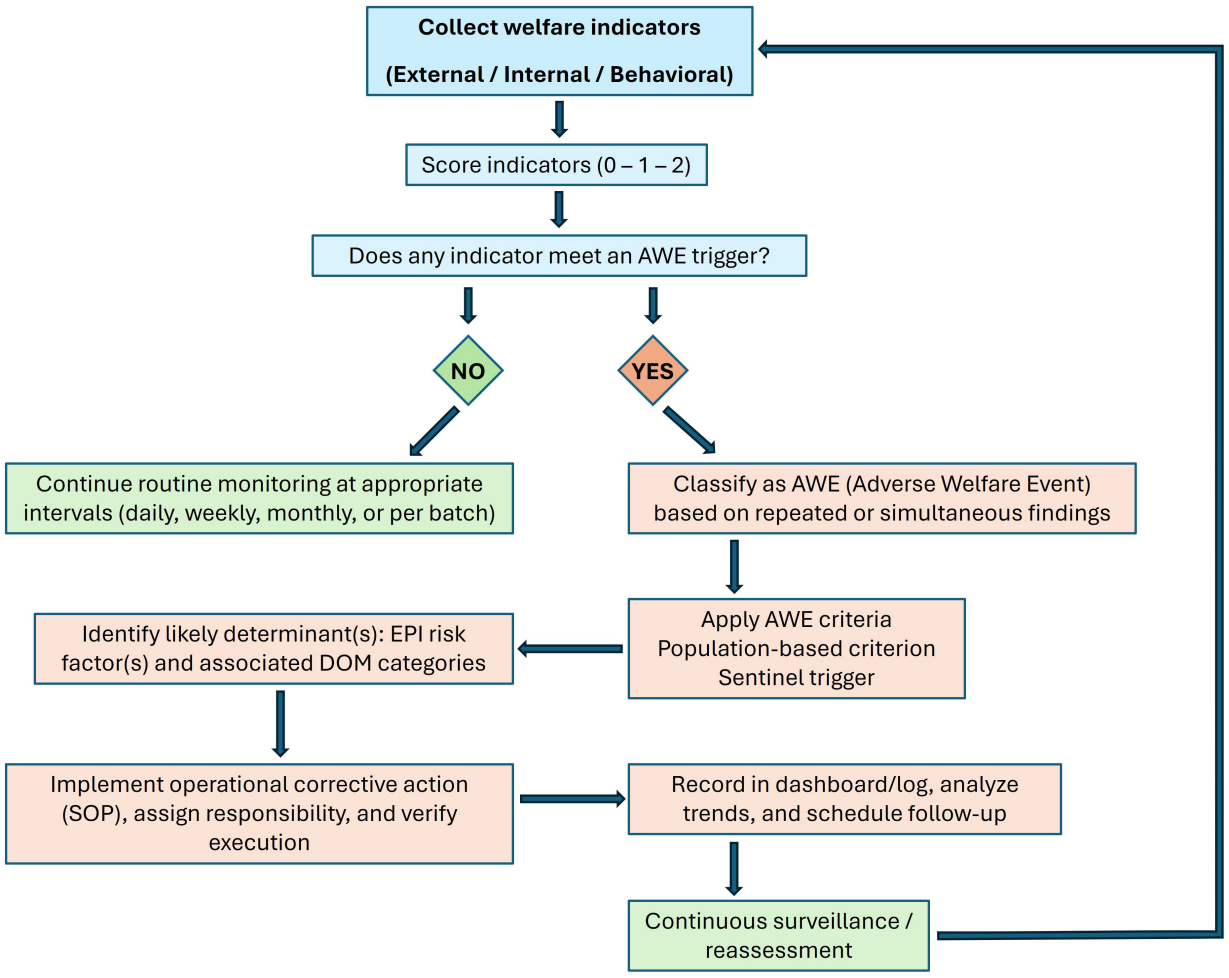
EPI-DOM operational workflow for welfare decision-making. Indicators are collected and scored (0–2). When an AWE trigger is met, population-based and sentinel criteria are applied to classify the event and link findings to EPI risk factors and associated DOM categories. SOP-based corrective actions are implemented, documented, and followed by continuous surveillance and reassessment.

Interpretation uses three operational elements: a population-based criterion, a sentinel criterion, and an operational corrective action. The population-based criterion distinguishes expected variation from meaningful deviation at group level, while the sentinel criterion functions as an early-warning trigger conceptually analogous to epidemiological surveillance thresholds (66). Corrective actions are derived from the implicated domain categories (DOM; Figure 1) and specify practical, proportional, and verifiable responses. The decision-making workflow from scoring to AWE detection, linkage to EPI/DOM, and SOP-based corrective actions is summarized in Figure 3.

#### 3.4.4 Farm-level sampling for AWEs: prevalence estimation and sentinel detection

On farms holding thousands of fish, AWE assessments cannot be conducted as a census and must rely on representative sampling. Within EPI-DOM, two epidemiological objectives are distinguished: (i) estimating AWE frequency (prevalence/proportion) in a pond or batch, and (ii) early detection of AWE presence with high probability (sentinel sampling). For both objectives, classical veterinary epidemiology approaches are applied (66).

**a) Population-level AWE: estimating prevalence (proportion) with defined precision**

When the goal is to estimate what fraction of a pond shows an AWE (e.g., fin erosion, skin lesions, overt deformities), the sample size for a proportion is calculated as:

**Sample size to estimate a proportion**


$$\begin{array}{l} n_{0}=\frac{Z^{2}P_{\exp}\left(1-P_{\exp}\right)}{d^{2}}\; \end{array}$$ (1)

where *Z* is the Z value for the desired confidence level (95%: 1.96; 99%: 2.576), *P*_exp_is the expected prevalence (if unknown, 0.5 is used as the most conservative scenario), and *d* is the desired absolute precision (maximum tolerated error, expressed as a proportion; e.g., 0.10 = ±10%) (67).

If the pond population size is known (*N*), a Finite population correction can be applied (when *n*_0_is large relative to *N*) (67):

**Finite population correction**


$$\begin{array}{l} n_{adj}=\frac{n_{0}}{1+\frac{\left(n_{0}-1\right)}{N}}\; \end{array}$$ (2)

**Practical example (pond with** *N* = 10, 000 **fish**): using *P*_exp_ = 0.5 (conservative) and an operational precision of *d* = 0.125 (±12.5%; useful for rapid field diagnosis):

- **95% (Z** **=** **1.96)**: *n*_0_ = 61.47⇒*n*_*adj*_≈61.10⇒ sample **62 fish**
- **99% (Z** **=** **2.576)**: *n*_0_ = 106.17⇒*n*_*adj*_≈105.07⇒ sample **106 fish**

**Operational note:** if higher precision is required (e.g., *d* = 0.10, ±10%), sample size increases substantially (≈96 at 95% and ≈166 at 99% before correction; for large *N* the finite-population adjustment is typically modest), which is often impractical during farm visits. *n*_*adj*_ denotes the finite-population adjusted sample size.

**b) Sentinel sampling: sample size to “detect at least one case” (presence/absence)**

When the objective is sentinel surveillance (i.e., “to be reasonably confident that, if the problem exists above a minimum frequency, we will observe it”), the calculation is framed as the probability of detecting ≥1 affected fish if the true prevalence is at least a **design prevalence** (p^*^). For large populations, a widely used binomial approximation is:

**Detecting** **≥1 case given a design prevalence** p^*^


$$\begin{array}{l} n=\frac{\ln\left(1-CL\right)}{\ln\left(1-p^{*}\right)}\; \end{array}$$ (3)

where CL is the desired confidence level (0.95 or 0.99) and p^*^ is the minimum prevalence, one intends “not to miss” (e.g., 0.05 = 5%) (67).

**Example** (p^*^ = 5%):

- **95%**: *n* = ln(0.05)/*ln*(0.95) = 58.4⇒ **59 fish** (≈60)
- **99%**: *n* = ln(0.01)/*ln*(0.95) = 89.8⇒ **90 fish**

**Key interpretation**: this is where the “≈60 fish” rule naturally emerges—sampling ~60 fish yields ~95% probability of observing at least one fish with the AWE if the true pond-level prevalence is ≥5%.

**c) Two-stage strategy (external vs. internal indicators)**

A two-stage approach is typically efficient during handling events (e.g., grading). First, define an external-indicator subsample (*n*_e*xt*_) using (a) for prevalence estimation or (b) for sentinel detection (typical recommendation: 60 fish/pond at 95% or 90 fish/pond at 99% for p^*^). Second, randomly select an internal-indicator subsample (*n*_*int*_) from the same sampling frame (same pond, date, and context), constrained by cost/time and procedure biology. When historical farm information exists, p^*^ should be selected based on prior evidence; conservative sentinel surveillance uses the lowest operationally relevant p^*^ to avoid under-sampling (66, 67).

#### 3.4.5 Indicator synthesis and cluster-level interpretation

To construct reference values and operational thresholds, we conducted a systematic integration of data from experimental tilapia studies, clinical reports, and hematological and biochemical ranges widely accepted in aquaculture, as well as population-level observations under commercial and laboratory conditions. Because of inherent variability associated with age, temperature, nutrition, and culture system, values were harmonized using a comparative epidemiological criterion, prioritizing those obtained under controlled conditions and with consistent analytical methodologies. These ranges were incorporated into the EPI-DOM framework by identifying the affected domain (DOM), particularly within Management, which enabled each physiological deviation to be translated into a concrete operational risk. Thus, the guide does not only present normal and abnormal values, but also link each deviation to its causal explanation, its classification as a sentinel AWE when applicable, and the corresponding operational corrective action aligned with prevention, risk control, and continuous welfare improvement principles. This integration ensures that management decisions are grounded in robust physiological evidence and transparent epidemiological criteria.

#### 3.4.6 Operational indicators and severity scale

The EPI-DOM framework integrates direct observation of the animal with analysis of management, environment, and interaction conditions (20), enabling qualitative information to be transformed into quantitative, comparable, and actionable data. In this guide, we propose that each indicator—external and internal—be evaluated using a **0–2 severity scale**, designed to reflect event intensity and its potential impact on individual or population welfare.

#### 3.4.7 Operational clusters within the EPI-DOM framework

Information derived from individual indicators is translated into operational clusters representing the fish's functional state across three welfare dimensions: external, internal, and behavioral. These clusters enable comparisons among batches, ponds, treatments, caretakers, or management conditions using a common metric, strengthening traceability and the epidemiological orientation of the model.

Three main clusters are defined:

- External Indicators Cluster (EIC)
- Internal Indicators Clusters (IIC)
- Behavioral Indicators Cluster (BIC)

**a) External Indicators Cluster (EIC)**

EIC evaluates visible alterations related to the fish's structural and biomechanical integrity: skin, fins, eyes, opercula, body condition, lesions, and morphological signs compatible with AWEs. For each evaluated individual, the calculation is:


$$\begin{array}{l} EIC=\frac{\sum Pi}{n}\; \end{array}$$ (4)

where *P*_*i*_ is the score assigned to each indicator (0 = normal, 1 = alteration, 2 = consolidated AWE) and *n* is the total number of indicators assessed. The interpretation of the cluster result is shown in Table 2.

**Table 2:** Cluster interpretation.

Range	Interpretation	Action
< 0.5	Stable welfare	Routine monitoring
0.5–1.1	Mild to moderate risk	Preventive review within 48 h
≥1.2	Consolidated AWE	Immediate intervention and follow-up until resolution

**b) Internal Indicators Cluster (IIC)**

IIC evaluates physiological and biochemical responses to homeostatic disruption: glucose, lactate, cortisol, osmolality/electrolytes, plasma proteins, tissue-damage markers, oxidative stress markers, among others. Because these indicators differ in physiological sensitivity, their combination may require standardization through weighting (W) for advanced or comparative analyses. The base formula is analogous:


$$\begin{array}{l} IIC=\frac{\sum Pi}{n}\; \end{array}$$ (5)

The IIC enables precise identification of the physiological magnitude of an adverse event and is essential for intensive systems and controlled anesthesia, sedation, and experimental protocols.

**c) Behavioral Indicators Cluster (BIC)**

The BIC integrates indicators related to:

- Species-typical behavior: swimming patterns, feeding motivation, space use.
- Conspecific interactions: aggression, dominance hierarchies, shoal cohesion, avoidance.
- Human interaction: shyness, habituation, response to handling or disturbance.
- Mental state: apathy, fear, lethargy, erratic swimming, vigilance.

Due to ethological complexity, intra- and inter-individual variability, and the need for repeated observations and sequential analyses, the BIC uses a hierarchical structure. This manuscript intentionally introduces the BIC at an operational–conceptual level, because rigorous behavioral welfare assessment requires standardized observation protocols (ethogram definition, sampling design, observer training and reliability), repeated measures, and context-specific interpretation. The full methodological development of the BIC (hierarchical substructure, species-specific scales, and advanced analyses) will therefore be presented as a dedicated second contribution to ensure methodological depth comparable to that provided here for external and internal indicators and to avoid superficial treatment.

**d) Diagnostic integration**

The three clusters can be assessed independently or integrated into a welfare diagnostic matrix able to:

- identify the origin of the AWE (physical, physiological, or behavioral),
- link the event to the affected EPI-DOM domain (Management, Environment, or Interaction),
- define the corresponding operational corrective action, and
- support management decisions based on epidemiological evidence.

An AWE is defined when:

- at least one indicator has a score **≥2**, or
- a cluster reaches a value **≥1.2**, reflecting an aggregated problem.

Longitudinal monitoring enables detection of trends, anticipation of systemic failures, prioritization of risk factors, and documentation of interventions in operational manuals, strengthening traceability and continuous welfare improvement in aquaculture (66, 68, 69).

#### 3.4.8 Operational welfare indicators in Tilapia (external and internal)

Below, operational animal welfare indicators are presented grouped by response type (external and internal). Each indicator includes: (i) the observable manifestation (what to assess and how to recognize it), (ii) linked risk factor(s) (EPI), and (iii) the management categories/domains (DOM) where preventive or corrective action should be implemented.

### 3.5 External indicators in tilapia

External indicators constitute the first line of welfare assessment because they reflect visible alterations in body integrity, the tegument, fins, and other external structures. These manifestations represent the observable expression of the individual's physiological and health status in response to risk factors (EPI) present in the production environment, enabling non-invasive detection of injuries, parasitism, nutritional imbalances, or adverse environmental conditions. Within the EPI-DOM framework, external indicators are analyzed as observable events that capture the fish's biological response to the interaction among the Management, Environment, and Interaction domains. Their systematic assessment provides objective information to infer welfare status and to establish action priorities, both for preventing adverse events and for correcting critical situations (20). The indicators presented below were selected based on their applicability in commercial and experimental Tilapia culture systems. Each is accompanied by an operational description, associated risk factors (EPI), implicated management categories (DOM), an individual scoring system (Pi), a population-based criterion, a sentinel AWE criterion, and operational corrective actions. For rapid field screening and reader navigation, a schematic overview of all external indicators (EPI drivers, DOM action points, and sentinel criteria) is provided in Table 3.

**Table 3:** External indicators cluster (EIC)—operational overview for rapid screening (EPI-DOM).

Indicator	What to look for	Dominant EPI drivers (keywords)	Priority DOM categories (where to act)	Sentinel AWE rule	First-line corrective action
3.5.1. Scale loss/cutaneous erosion	Detached scales, erythema/erosion/abrasion, inflamed exposed skin	Parasites; bacterial coinfections (Aeromonas); rough handling; high density; poor water (DO/NH3/T°/salinity); contaminants	Animal (pop); Health-sanitary; Therapeutics; Natural resources (water); Human resources	≥2 fish with Pi = 2	Reduce density/handling; verify DO–NH_3_-T°; adjust grading; activate health surveillance
3.5.2. Cutaneous hemorrhages/petechiae	Pinpoint/diffuse red spots (petechiae) ± edema/systemic compromise	Bacterial (Aeromonas/Strep); viral (TiLV/TiPV); coinfections; poor water; handling/transport stress; dysbiosis	Health-sanitary; Natural resources (water); Animal (pop); Nutrition-feeding	≥2 fish with Pi = 2	Verify DO–NH_3_–${\text{NO}}_{2}^{-}$–T°-pH; suspend stressors; diagnostics; biosecurity; monitor 72 h
3.5.3. Mouth lesions (lips/jaw)	Cuts/erosions/inflammation of lips/jaw; feed capture difficulty	Collisions (walls/flows); abrasive nets; rough handling; competition (high density/poor feed distribution); poor water increasing collision risk	Animal (pop/ind); Nutrition-feeding; Human resources	≥2 fish with Pi = 2	Remove abrasive edges/adjust flows; optimize feed distribution/pellet size; reduce competition; train staff
3.5.4. Operculum integrity	Opercular tearing/asymmetry/folding/shortening; gill exposure; altered opercular movement	Genetic/QTL predisposition; contaminants (metals/oil/dyes); chronic environmental stress/variability	Genetics-reproduction; Animal (pop/ind); Natural resources (water); Economic-productive	≥2 fish with Pi = 2	Improve water quality/contaminant control; record prevalence; exclude severe defects from broodstock
3.5.5. Fin integrity	Frayed/eroded fins; notches/tissue loss; bleeding; locomotion impairment (advanced)	High density; aggression; poor feeding rate; nutritional deficiencies; poor water (NH_3_/${\text{NO}}_{2}^{-}$/DO); rough handling/transport	Animal (pop/ind); Nutrition-feeding; Natural resources (water); Transport-logistics; Human resources	≥2 fish with Pi = 2	Reduce density; mitigate aggression; adjust diet; improve DO and reduce NH_3_/${\text{NO}}_{2}^{-}$; review handling/transport
3.5.6. Superficial ulcers/abscesses	Open ulcerative lesions ± exudate/necrosis; inflammation, lethargy	Bacterial (Aeromonas/Strep/Flavobacterium); opportunistic fungi; wounds from handling; chronic stress (water/density/transport)	Health-sanitary; Transport-logistics; Human resources; Therapeutics	≥2 fish with Pi = 2	Correct water; diagnostics; reduce density/handling; reinforce biosecurity; therapy per susceptibility/regulations
3.5.7. Visible deformities	Spinal/jaw/fin deformities affecting swimming/feeding/performance	Genetics/inbreeding; mineral/vitamin deficits; chronic poor water; contaminants/metals; chronic stress (T°/salinity fluct.)	Genetics-reproduction; Nutrition-feeding; Natural resources (water); Health-sanitary	≥2 fish with Pi = 2	Review diet (Vit C, Ca, P); water history; chronic stressors; genetic control; cull severe; assess contaminants
3.5.8. Ocular integrity	Exophthalmia, opacity, hemorrhage, partial/total eye loss	Streptococcus/other bacteria; handling trauma; hypoxia/poor water; hyperoxia/fluctuations	Health-sanitary; Transport-logistics; Human resources; Natural resources (water)	≥2 fish with Pi = 2	Verify DO/NH_3_/${\text{NO}}_{2}^{-}$; revise handling/transport; presumptive strep diagnostics; isolate; reduce density/handling
3.5.9. Caudal peduncle integrity	Erosions/thinning/discoloration; reduced propulsion, fatigued swimming	Excessive currents/flow velocity; high density abrasion; handling/transport trauma	Animal (pop); Infrastructure/hydraulics (Env); Transport-logistics; Human resources	≥2 fish with Pi = 2	Adjust density/biomass; correct flows/deflectors/surfaces; fix handling to avoid caudal compression; reduce handling
3.5.10. Body condition (Fulton's K)	Visual thin/normal/robust + Fulton's K deviation vs historical/mean (Kμ)	Underfeeding/poor delivery; overfeeding/energy excess; chronic disease; chronic stress/water/density	Nutrition-feeding; Health-sanitary; Economic-productive	≥2 fish with Pi = 2	Review ration/distribution; adjust protein and feeding rate by stage; rule out chronic/parasitic disease; improve environment
3.5.11. Visible external parasitosis	Visible parasites/cysts/plaques; mucus; rubbing; ventilatory distress	Biosecurity/quarantine failures; poor water; infected stock/movements; high transmissibility	Health-sanitary; Natural resources (water); Animal (pop); Economic-productive	≥2 fish with Pi = 2	Treat per parasite group + correct DO/NH_3_/${\text{NO}}_{2}^{-}$/solids; quarantine/disinfection; reduce density; seasonal monitoring

#### 3.5.1 Scale loss/cutaneous erosion

Areas with scales partially or completely detached; exposed skin with visible erythema, erosions, abrasions, or inflammation, reflecting evident and readily identifiable tegument damage (70–72).

**EPI** → Parasitic infections, environmental stressors, and nutritional deficiencies (73). Bacterial infections, particularly *Aeromonas hydrophila* and coinfections (74). Poor handling, rough manipulation, or high stocking density that increase abrasion and stress (71, 75, 76). Suboptimal water conditions (hypoxia, ammonia, temperature, or salinity out of range) predisposing to tegument damage (77, 78). Chemical exposure and environmental contaminants (73, 79–81).

**DOM**→**Animal (population)**: adjust stocking density and handling protocols (71). **Health and sanitary**: surveillance, diagnosis, and vaccination (82, 83). **Therapeutics**: prudent use of antimicrobials and probiotics (52, 83). **Natural resources (water -environment domain-):** control dissolved oxygen (DO), ammonia, and temperature through biofloc or RAS systems (53, 56). **Human resources**: continuous training in good practices (71, 75).

**Individual scoring system (Pi)**

**0**: intact tegument, no areas of scale loss.

**1**: localized and superficial scale loss, without bleeding or functional compromise.

**2**: extensive or multifocal scale loss with bleeding, ulceration, or associated signs of stress.

**Population-based criterion**

If **>20%** of sampled fish have **Pi** **≥** **1**, population-level tegument deterioration is considered present and intervention is required.

**Sentinel AWE criterion**

If the sample includes **≥2 fish with Pi** **=** **2**, an **AWE** is considered present, regardless of the total batch percentage or pond size.

**Operational corrective action**

Immediately reduce stocking density and handling intensity; review water-quality parameters (DO, NH_3_, temperature); adjust capture/grading procedures; and activate health surveillance if coinfection is suspected.

#### 3.5.2 Cutaneous hemorrhages/petechiae

Small pinpoint or diffuse red spots on the skin, visible to the naked eye, indicative of superficial vascular damage associated with health-related, environmental, or handling stressors (84–86).

**EPI** → Bacterial infections (*Aeromonas* spp., *Streptococcus agalactiae*) (85, 87, 88). Viral infections (TiLV/TiPV) (84, 87, 89). Coinfections (83, 90). Poor water quality (91, 92). Handling/transport stress (93). Dysbiosis (70, 72).

**DOM**→**Nutrition and feeding**: functional diets and antioxidant support (56, 94, 95). **Health and sanitary**: early diagnosis, outbreak control, and vaccination (96–98). **Animal (population)**: appropriate stocking density and safe transport (93). **Natural resources (water -environment domain-):** dissolved oxygen, ammonia/nitrite, and temperature control (91).

**Individual scoring system (Pi)**

**0**: no visible hemorrhages; normal skin coloration and vascular pattern.

**1**: localized petechiae or small hemorrhagic areas (approximately < 1 cm^2^), without edema or obvious systemic signs.

**2**: extensive or multifocal hemorrhages, with possible edema, associated hemorrhagic lesions, or signs of generalized compromise (lethargy, abnormal swimming, hyperventilation).

**Population-based criterion**

If >**15%−20%** of sampled fish have **Pi** **≥** **1**, population-level hemorrhagic compromise is considered present and intervention is required.

**Sentinel AWE criterion**

If the sample includes **≥2 fish with Pi** **=** **2**, an **AWE** is considered present, regardless of the total batch percentage or pond size.

**Operational corrective action**

Immediately verify water quality: DO, NH_3_, ${\text{NO}}_{2}^{-}$, temperature, and pH. Reduce stressors: decrease stocking density when feasible, avoid unnecessary handling, and suspend grading/transfers. Activate health diagnostics: collect samples for bacteriology/virology if septicemia is suspected (*Aeromonas, Streptococcus*, TiLV, coinfections). Reinforce biosecurity: disinfect equipment, restrict movements between units, and control entries/exits. Nutritional support: implement functional diets (immunostimulants/antioxidants) when appropriate for the context. Intensified monitoring for 72 h: daily follow-up of lesion progression and mortality.

#### 3.5.3 Mouth lesions (lips, jaw)

Visible cuts, erosions, or inflammation of the lips and jaw, including focal hemorrhage, irregular margins, and difficulty capturing feed (99–101).

**EPI** → Impacts against walls, corners, or inappropriate water flows (99, 100). Abrasive nets and rough handling during capture/grading (76, 101). Feed competition due to high stocking density or poor feed distribution (102, 103). Suboptimal water quality that increases collision risk (99, 100).

**DOM**→**Animal**: populations placed in unsuitable tanks/ponds; review operational conditions that promote collisions; consider redesigning edges, smoothing surfaces, and adjusting water flows (100, 104). **Nutrition and feeding**: scheduling of feeding times and rations; pellets appropriate for body size (102, 105). **Human resources**: training in low-trauma capture and grading using appropriate nets (76, 101).

**Individual scoring system (Pi)**

**0**: intact mouth, no visible erosion, cuts, or inflammation.

**1**: localized lesion (mild erosion/cut) without obvious difficulty feeding.

**2**: extensive or deep lesion with inflammation, bleeding, or observable difficulty capturing feed.

**Population-based criterion**

If **>20%** of sampled fish have **Pi** **≥** **1**, population-level oral deterioration is considered present and intervention is required.

**Sentinel AWE criterion**

If the sample includes **≥2 fish with Pi** **=** **2**, an **AWE** is considered present regardless of the overall percentage or pond size.

**Operational corrective action**

Inspect infrastructure: remove abrasive edges and aggressive surfaces. Adjust feeding method: ensure homogeneous distribution and pellet size appropriate to fish size. Reduce competition: adjust stocking density and increase/redistribute feeding points. Train personnel: implement low-trauma capture/handling practices. Daily visual monitoring for ≥72 h post-correction.

#### 3.5.4 Operculum integrity

Tearing of the opercular margin, asymmetry, folding, or shortening; partial absence of the gill cover with gill exposure and altered opercular movement (106–109).

**EPI** **→** Genetic factors, inherited predisposition, and loci/QTLs associated with opercular malformations (106–109). Environmental contaminants (heavy metals, crude oil, and azo dyes) causing structural gill damage (110–114). Chronic environmental stressors and variability that compromise gill tissues (108, 113).

**DOM**→**Genetics and reproduction**: selection of broodstock and lines with lower prevalence of opercular defects (106–109). **Animal (individual and population):** batch-level prevalence monitoring, culling of severely affected individuals, and adjustment of rearing strategies (106, 108). **Natural resources**: water-quality management and reduction of contaminants/heavy metals that affect gill tissues (110–114). **Economic and productive**: incorporating opercular defect frequency into performance analyses and economic loss estimates (106, 108).

**Individual scoring system (Pi)**

**0**: opercula intact, symmetric, and functional.

**1**: mild asymmetry, irregular opercular margin, or partial folding without gill exposure.

**2**: tearing, marked shortening, or partial absence with gill exposure and/or clear alteration of opercular movement.

**Population-based criterion**

If **>10%** of sampled fish have **Pi** **≥** **1**, population-level opercular alteration is considered present and corrective intervention is required (genetics, environment, or management).

**Sentinel AWE criterion**

If the sample includes **≥2 fish with Pi** **=** **2**, an AWE is considered present due to potential respiratory compromise, regardless of the total batch percentage or pond size.

**Operational corrective action**

Review and adjust water quality to reduce gill damage risk. Assess infrastructure materials and tank/pond surfaces that may be causing trauma. Record prevalence and exclude broodstock with severe defects. Investigate environmental contaminants if lesions are frequent or progressive.

#### 3.5.5 Fin integrity

Frayed or eroded fins with irregular margins, petechiae or bleeding in fin rays and membranes, and tissue loss that may appear as notches or missing portions; in advanced cases, these changes are accompanied by impaired locomotion and reduced propulsive efficiency during swimming (115, 116).

**EPI** **→** Inappropriate feeding rate (excess or deficit) increasing competition and stress (115). Social aggression associated with unstable dominance hierarchies (117, 118). High stocking density increasing friction, aggression, and susceptibility to injury (116, 119–121). Nutritional deficiencies due to diets lacking adequate balance of protein, fatty acids, and micronutrients (80, 81, 122–124). Poor water quality with elevated ammonia/nitrite and low dissolved oxygen (71, 99, 100, 125, 126). Rough handling during grading or biometric procedures (71, 101).

**DOM** **→** **Nutrition and feeding:** adjustment of formulations, feeding rate, and functional additives (80, 81, 115, 123, 124, 127). **Animal management (individual and population):** regulation of stocking densities, size grading, and reduction of social aggression (71, 116, 119, 121). Handling protocols using appropriate nets to minimize trauma (71, 101). **Natural resources (water -environment domain-):** continuous monitoring of dissolved oxygen, ammonia, nitrite, and water exchange (99, 100, 125, 126). **Transport and logistics:** control of densities, oxygenation, and time during harvest, intra-farm transfers, and transport to slaughter (93).

**Individual scoring system (Pi)**

**0:** fins intact; no erosion, bleeding, or tissue loss.

**1:** irregular margins, small notches, mild erosion, or petechiae without substantial structural loss.

**2:** marked tissue loss, exposed fin rays, evident bleeding, or visible impairment of locomotion.

**Population-based criterion**

If **>20%** of sampled fish have **Pi** **≥** **1**, population-level fin integrity deterioration is considered present and may be associated with social, nutritional, or environmental stressors.

**Sentinel AWE criterion**

If the sample includes **≥2 fish with Pi** **=** **2**, an **Adverse Welfare Event (AWE)** is considered present due to sanitary risk and functional impairment, regardless of batch size.

**Operational corrective action**

Size-grade fish, reduce stocking density, and mitigate competition/aggression. Adjust diet (quality, rate, and frequency). Improve DO and reduce ammonia/nitrite. Review handling procedures and replace abrasive nets. Reassess transport conditions and handling/exposure times.

#### 3.5.6 Superficial ulcers/abscesses

Open lesions with erythematous margins and whitish or reddish plaques, with necrotic tissue and/or exudate; they may be accompanied by evident inflammation, pain, and lethargy, affecting both external appearance and behavior (128, 129).

**EPI** **→** Bacterial infections, primarily caused by *Aeromonas* spp., *Streptococcus* spp., and *Flavobacterium columnare* (128–131). Opportunistic fungal infections colonizing wounds (132). Rough handling and poorly healed injuries that serve as entry points for pathogens (92, 133, 134). Chronic stress associated with poor water quality, high stocking density, or transport (92, 135, 136).

**DOM** **→** **Health and sanitary:** surveillance and early diagnosis, water-quality control, vaccination and immunostimulation; cleaning and disinfection to interrupt transmission (76, 133, 137, 138). **Transport (including internal transfers):** planning of fasting, time, and adequate oxygenation; density control and correct handling during movements (134–137). **Human resources:** training in capture and restraint techniques, wound management, removal of mortalities, and compliance with welfare SOPs (92, 101, 133, 134). **Therapeutics:** treatment guided by antimicrobial susceptibility testing and local regulations (when applicable).

**Individual scoring system (Pi)**

**0:** intact skin; no open lesions or ulcerated areas.

**1:** localized, superficial ulcer/erosion without pus or evident necrosis.

**2:** moderate to severe, multifocal ulceration with pus, necrotic margins, or marked inflammation.

**Population-based criterion**

If **>10%** of sampled fish have **Pi** **≥** **1**, population-level tegument compromise is considered present and intervention is required (lower threshold due to sanitary risk).

**Sentinel AWE criterion**

If the sample includes **≥2 fish with Pi** **=** **2**, an **Adverse Welfare Event (AWE)** is considered present regardless of population size.

**Operational corrective action**

Review and correct water quality (DO, NH_3_, ${\text{NO}}_{2}^{-}$, temperature). Initiate bacterial diagnostics (*Aeromonas, Streptococcus*, etc.). Reduce stocking density and minimize handling. Inspect and modify nets, surfaces, and abrasive edges. Isolate affected lots and reinforce biosecurity. Consider therapy guided by susceptibility testing and local guidelines.

#### 3.5.7 Visible deformities

Externally detectable morphological alterations, including spinal deviations (lordosis, scoliosis, and kyphosis), mandibular malformations, and twisted or asymmetric fins, which impair locomotion, feeding, and productive performance (139–143).

**EPI** **→** Genetic factors, inbreeding, mutations, and lines with inherited predisposition (142, 144). Nutritional factors, including mineral and vitamin deficiencies, particularly calcium, phosphorus, and vitamin C (140, 145). Chronic poor water quality with altered dissolved oxygen, pH, and waste accumulation (146, 147). Pollution and prolonged exposure to heavy metals or aquatic contaminants (148–150). Sustained environmental stress, including critical fluctuations in temperature and salinity that affect skeletal development (151, 152).

**DOM** **→** **Genetics and reproduction:** broodstock selection, inbreeding control, and genetic line monitoring (142, 143, 153). **Feeding and nutrition:** diet adjustment to ensure essential minerals and vitamins for skeletal development (140, 145). **Health and sanitary:** early diagnosis of malformations and control of agents that compromise skeletal development (148, 154). **Natural resources:** continuous water-quality monitoring and reduction of contaminants and heavy metals (141, 146).

**Individual scoring system (Pi)**

**0**: normal morphology; no visible external deformities.

**1**: mild, localized deformity (spine, jaw, or fins) without obvious functional impairment.

**2**: moderate to severe deformity impairing locomotion and/or feeding; marked spinal curvature, severe fin twisting, or functionally relevant mandibular alteration.

**Population-based criterion**

If **>10%** of sampled fish have **Pi** **≥** **1**, a population-level skeletal development problem is considered present and intervention is required.

**Sentinel AWE criterion**

If the sample includes **≥2 fish with Pi** **=** **2**, an **Adverse Welfare Event (AWE)** is considered present regardless of batch size.

**Operational corrective action**

Review the batch nutritional history (vitamin C, Ca, and P). Evaluate water quality (DO, pH, chronic waste loads). Review stocking density records and prolonged stressors. Implement genetic selection and cull severely affected individuals. Assess potential exposure to heavy metals or other contaminants.

#### 3.5.8 Ocular integrity

Exophthalmia (protrusion of the eyeball), corneal opacity, intraocular hemorrhages, or partial/total loss of the eye, all visible to the naked eye and associated with pathological processes or mechanical damage (86, 155–158).

**EPI** **→** Bacterial infections: *Streptococcus* spp. and other agents associated with exophthalmia, opacity, and hemorrhage (86, 155, 159). Trauma during handling, capture, grading, and transfer causing ocular injury (156, 160). Chronic hypoxia or poor water quality increasing susceptibility and ocular outbreaks (161, 162). Hyperoxia and oxygen fluctuations linked to inadequate water management and physiological stress (163, 164).

**DOM** **→** **Health and sanitary:** early diagnosis, vaccination against *Streptococcus*, outbreak surveillance, and prudent antimicrobial use (83, 155, 165). **Transport and logistics:** reduce stress and impacts during transfer to prevent ocular lesions (137, 156). **Human resources:** training in low-stress capture, sampling, and grading protocols to prevent ocular damage (133, 134). **Natural resources:** control of DO and nitrogenous compounds (NH_3_/) and overall system stability.

**Individual scoring system (Pi)**

**0**: eyes intact; no exophthalmia, opacity, or hemorrhage.

**1**: mild lesion—partial opacity, mild protrusion, or focal hemorrhage without obvious functional compromise.

**2**: moderate to severe lesion—marked exophthalmia, total opacity, extensive hemorrhage, or partial/total loss of the eye.

**Population-based criterion**

If **>10%** of sampled fish have **Pi** **≥** **1**, a population-level ocular integrity problem is considered present and intervention is required.

**Sentinel AWE criterion**

If the sample includes **≥2 fish with Pi** **=** **2**, an **Adverse Welfare Event (AWE)** is considered present regardless of pond or batch size.

**Operational corrective action**

Immediately review critical water-quality parameters (DO, NH_3_, ${\text{NO}}_{2}^{-}$). Verify capture, grading, and transport protocols. Conduct presumptive diagnostics for streptococcosis (smear, culture, or qPCR if available). Isolate affected units, reduce stocking density, and minimize handling. Implement biosecurity measures and evaluate the need for treatment in accordance with local regulations.

#### 3.5.9 Caudal peduncle integrity

Visible lesions or erosions, progressive thinning of the caudal peduncle, discoloration, and reduced motor strength, expressed as fatigued swimming and diminished propulsion (73, 166, 167).

**EPI** **→** Swimming overload due to strong currents: jets or flow velocities poorly adjusted in RAS/BFT systems (52, 53). Excessive stocking density leading to increased contact, abrasion, and stress that compromise caudal skin and musculature (168). Physical trauma during handling and transport, including injuries from capture, grading, and transfers (137).

**DOM** **→** **Animal (population):** adjustment of stocking density and biomass, and spatial redistribution of fish (168). **Infrastructure/hydraulic design (environment domain):** pond/tank hydraulic design accounting for flow velocity, inlet orientation and deflectors, and non-abrasive surface materials (52, 53). **Transport and logistics:** implementation of SOPs and equipment that minimize impacts or compression of the caudal region (133, 134, 137). **Human resources:** SOPs for handling and internal movements.

**Individual scoring system (Pi)**

**0**: peduncle intact; no erosions and no locomotor alterations.

**1**: mild lesion—superficial erosion, slight discoloration, or mild fatigue without marked loss of propulsion.

**2**: moderate to severe lesion—deep erosion, evident thinning, partial tissue loss, or clear propulsion impairment (fatigued swimming, difficulty advancing).

**Population-based criterion**

If **>15%** of sampled fish have **Pi** **≥** **1**, a population-level caudal peduncle integrity problem is considered present.

**Sentinel AWE criterion**

If the sample includes **≥2 fish with Pi** **=** **2**, an **Adverse Welfare Event (AWE)** is considered present regardless of pond size.

**Operational corrective action**

Adjust stocking density and redistribute biomass. Review currents, flow rates, deflectors, and surface materials. Correct handling and transport practices to prevent impacts and caudal compression. Minimize handling until lesions are controlled and evaluate risk of secondary infection.

#### 3.5.10 Body condition (Fulton's K)

Body condition is evaluated through visual inspection (sunken abdomen = thin; balanced proportions = normal; overall bulking = robust) and by calculating Fulton's condition factor (K) from body weight and length (102, 115, 169). Use of historical K values from the production unit—or the mean K of individuals within a pond or batch—is recommended to identify fish that deviate above or below the expected range or the population mean (Kμ) (115).

**EPI** **→** Underfeeding: deficient diets or insufficient feed delivery (115, 170, 171). Overfeeding: excessive rations or energy-dense formulations leading to obesity or visceral fat accumulation (59, 115, 172). Chronic disease and prolonged infectious/parasitic processes that reduce body condition (173). Prolonged stress, adverse environmental conditions, and inadequate management that impair K (71, 135).

**DOM** **→** **Feeding and nutrition:** diet quality, protein levels (30%−35%), and feeding rate (~5% BW/day) influence growth and efficiency (59, 102, 172), particularly under high stocking densities. **Health and sanitary:** control of chronic diseases, surveillance of bacterial/parasitic outbreaks, and reduction of environmental stressors (101, 135, 173). **Economic and productive:** K is directly related to feed conversion, profitability, and sustainability; both underfeeding and overfeeding generate economic losses (123, 174, 175).

**Individual scoring system (Pi)**

**0**: normal body condition—proportionate abdomen; K within the expected range for size/age or within ±10% of the batch/population mean (*K*_μ_).

**1**: mild abnormal condition—slightly sunken or slightly distended abdomen; K 10%−20% below/above *K*_μ_.

**2**: severe abnormal condition—K ≥20% below/above *K*_μ_, with marked emaciation (pronounced sunken abdomen) or clear excess visceral fat.

**Population-based criterion**

If **>20%** of sampled fish have **Pi** **≥** **1**, a population-level body-condition problem is considered present and intervention is required (nutritional, sanitary, and/or environmental).

**Sentinel AWE criterion**

If the sample includes **≥2 fish with Pi** **=** **2**, an **Adverse Welfare Event (AWE)** is considered present due to severe metabolic compromise, regardless of batch size.

**Operational corrective action**

Review ration size, frequency, timing, and feed distribution. Adjust dietary protein (30%−35%) and feeding rate (~5% pond biomass/day) according to production stage. Investigate chronic or parasitic disease if marked thinness is observed. Improve environmental parameters, reduce prolonged stressors, and reinforce biosecurity. Assess economic impact (feed conversion ratio and costs).

#### 3.5.11 Visible external parasitosis

Macroscopic presence of parasites attached to the skin and fins (*Argulus, Lernaea*); punctiform plaques or cysts on the skin; whitish nodules on the gills compatible with *Trichodina* or *Ichthyophthirius*; increased mucus production, rubbing against surfaces, and signs of ventilatory distress (176–179).

**EPI** **→** Poor biosecurity, lack of quarantine, or inadequate disinfection facilitates the introduction and transmission of ectoparasites (180, 181). Contaminated water or suboptimal parameters—elevated ammonia/nitrates and low dissolved oxygen—increase parasite prevalence (179, 182). Introduction of infected animals/translocation, movements between farms, and the use of fish without health control increase risk (183, 184).

**DOM** **→** **Health and sanitary:** routine diagnosis, approved antiparasitic control, and preventive protocols in hatcheries (180, 185). **Animal (individual and population):** density regulation and size grading to reduce transmission; seasonal monitoring of parasite burdens (186, 187). **Economic and productive:** compliance with sanitary standards and certifications to maintain market access and reduce outbreak-related economic losses (177, 188). **Natural resources (water -environment domain-):** correction of DO, NH_3_, ${\text{NO}}_{2}^{-}$, solids, and water exchange/stability.

**Individual scoring system (Pi)**

**0**: no visible parasites; skin, fins, and gills without cysts/plaques or excessive mucus.

**1**: mild presence–1–3 attached parasites (*Argulus/Lernaea*) or a few punctiform cysts on skin/gills compatible with early *Trichodina, Ichthyophthirius*, or monogeneans; moderate mucus.

**2**: moderate to severe infestation—>3 visible parasites, multiple white cysts/plaques on skin, congested gills, abundant mucus, repeated rubbing, and/or evident ventilatory distress.

**Population-based criterion**

If **>15%** of sampled fish have **Pi** **≥** **1**, established external parasitosis is considered present at the population level and sanitary intervention is required (treatment plus environmental correction).

**Technical note:** this threshold is lower than for other indicators because ectoparasites have high horizontal transmissibility.

**Sentinel AWE criterion**

If the sample includes **≥2 fish with Pi** **=** **2**, an **Adverse Welfare Event (AWE)** is considered present due to parasitosis, regardless of the total batch percentage or pond size.

**Operational corrective action**

Implement an **approved antiparasitic treatment** according to the target parasite group (*Argulus, Lernaea*, monogeneans, *Trichodina*). Review and correct critical parameters: DO, NH_3_, ${\text{NO}}_{2}^{-}$, solids, and water exchange. Implement quarantine and disinfection to prevent reinfection and reassess batch origin. Reduce density when feasible; size-grade if differential transmission is suspected. Establish seasonal monitoring of parasite burdens.

**Integration of proposed external indicators**

Integrating the proposed external indicators enables a structured assessment of tilapia physical and health status, facilitating early detection of visible alterations associated with management-, environmental-, or pathogen-related risk factors. Each indicator provides complementary information that, in combination, reflects the recent history of culture conditions and the effectiveness of biosecurity and management practices. Although some visual parameters, such as pigmentation or color changes, may provide contextual signals of acute stress or metabolic dysfunction, their high genetic and environmental variability limits their use as primary indicators within this framework. Therefore, they are treated as complementary observations that should be interpreted only in conjunction with other validated parameters. Overall, systematic evaluation of external indicators constitutes the first operational level of welfare diagnosis under the EPI-DOM model, and regular application provides the empirical basis for correlating physical observations with physiological and internal parameters in the next analytical level.

### 3.6 Internal indicators in tilapia

Internal welfare indicators in Tilapia are a key tool to assess the organism's **true physiological state** in response to environmental, handling, nutritional, and sanitary stressors. Unlike external or behavioral indicators, which reflect observable responses, internal indicators enable the detection of **subclinical alterations**, early adaptive processes, and transitions from homeostasis toward **chronic stress** or functional impairment. From a physiological perspective, these indicators integrate the coordinated responses of multiple systems—hematological, metabolic, respiratory, cardiovascular, endocrine, immune—acting together to sustain viability.

In tilapia, a species recognized for high physiological plasticity and tolerance to adverse conditions, many mechanisms can maintain vital functions even under substantial stress; however, this tolerance may **mask sustained physiological distress**, particularly when stressors are prolonged or overlapping. Internal indicators should not be interpreted as universal absolute values, but rather as **dynamic deviations from baseline**, dependent on production context, species/strain, age and size, environmental conditions, and analytical methods. Their value therefore lies less in rigid numeric cut-offs and more in the **physiological coherence of the observed pattern**, especially when assessed in an integrated and longitudinal manner.

Within the EPI-DOM framework, internal indicators serve a **confirmatory and explanatory** function: they link risk factors (EPI) to specific failures in management categories (DOM), evidencing the real physiological impact of operational decisions related to water quality, density, handling, transport, or therapeutics. In doing so, their analysis provides an objective basis for corrective decision-making, welfare-practice validation, and prevention of functional damage. Ultimately, the use of internal indicators strengthens welfare assessment by moving beyond a reactive approach centered on disease or mortality, and toward a **preventive, functional welfare** perspective—aimed not only at avoiding harm, but at sustaining adaptive capacity within physiologically safe and ethically responsible margins.

Within this guide, the values and cut-off points used for internal indicators should be understood as operational thresholds to support welfare and management decision-making, rather than as universal clinical reference intervals. In physiology, an operational range refers to the interval of values within which a biological system or physiological variable can function in a stable and adaptive manner to maintain homeostasis. It represents the safe functional limits within which the organism can adjust its responses without progressing toward dysfunction or pathology (246). Their interpretation depends on the production and analytical context, including farming system, water quality, temperature, salinity, stocking density, feeding practices, production stage, sanitary status, sampling protocol, and laboratory methodology. Therefore, each production unit is encouraged to establish and periodically update its own local baselines and operational ranges using clinically healthy fish and standardized sampling procedures. In all cases, internal indicators should be interpreted in an integrated manner together with external, behavioral, and management indicators, avoiding conclusions based on a single isolated parameter.

Internal indicators are extensive by necessity, because they translate welfare risk into measurable physiological consequences. To improve readability while preserving operational utility and reproducibility, we summarize the internal indicators in two tables. Tables 4, 5, provides a “core” operational overview (respiratory rate, heart rate, hematological and blood biochemistry indicators) intended for routine surveillance and first-line decision-making. Table 6 provides a laboratory-oriented module focused on tissue injury and oxidative stress (hepatic and muscle enzymes and redox biomarkers). The main text retains the physiological rationale and integrated interpretation within the EPI-DOM logic.

**Table 4:** Internal welfare indicators in tilapia—core operational summary (IIC-Core and hematological).

Indicator (IIC-Core and hematological)	Operational signal (what you measure/observe)	Main drivers (EPI keywords)	Main domains (DOM keywords)	Operational triggers (Pi/population/sentinel)	First-line corrective action (operational)
3.6.1. Respiratory rate (opercular ventilation; OPM)	OPM pattern vs batch baseline; rhythm/amplitude; gasping/asynchrony	Hypoxia; NH_3_/${\text{NO}}_{2}^{-}$; handling/transport	Water quality; Health/sanitary; Animal (pop); Transport; Human resources	**Pi 0** baseline pattern; **Pi 1** moderate tachy/bradypnea or irregularity vs baseline; **Pi 2** marked disturbance + persistent gasping/asynchrony. **Pop:** >20% Pi ≥ 1. **Sentinel:** ≥2 fish Pi = 2	Verify DO/T/NH_3_/${\text{NO}}_{2}^{-}$ immediately; restore aeration/exchange; suspend handling until normalization; reduce biomass if feasible; set stop-criteria at Pi ≥ 1; reassess 12–24 h
3.6.2. Heart rate (Doppler/ECG)	HR vs baseline; rhythm variability/irregularity	Severe/prolonged hypoxia; NH_3_ toxicity; handling stress; anesthesia/sedation	Water quality; Therapeutics; Transport; Health; Human resources	**Pi 0** stable baseline; **Pi 1** moderate tachy/brady vs baseline without arrhythmia; **Pi 2** sustained extreme tachy/brady + high variability/irregularity. **Pop:** >20% Pi ≥ 1. **Sentinel:** ≥2 fish Pi = 2	Correct DO/NH_3_ first; postpone invasive procedures; review anesthesia dosing/monitoring; standardize measurement timing; escalate metabolic panel if Pi = 2 persists
3.6.3.1. Hematocrit (Hct)	% RBC volume	Anemia/bleeding; toxins (e.g., Hg); osmotic stress; dehydration; TiLV	Water quality; Nutrition; Animal; Human resources	**Pi 0** 25–45%; **Pi 1** 20–24 or 46–50; **Pi 2** < 20 or >50. **Pop:** >20% Pi ≥ 1. **Sentinel:** ≥2 fish Pi = 2	Check NH_3_/${\text{NO}}_{2}^{-}$/DO and contaminants; verify salinity management; adjust density; review micronutrients (Fe/B12/folate/Cu); investigate infection/toxicity
3.6.3.2. Hemoglobin (Hb)	g/dl oxygen-carrying capacity	Heavy metals; chronic hypoxia; osmotic stress; bleeding/hemolysis	Water quality; Nutrition; Animal; Human resources	**Pi 0** 7–12; **Pi 1** 6–6.9 or 12.1–14; **Pi 2** < 6 or >14. **Pop:** >20% Pi ≥ 1. **Sentinel:** ≥2 fish Pi = 2	If low: investigate metals/hemolysis, improve diet (Fe/B12), biosecurity. If high: evaluate chronic hypoxia/density; aeration upgrade; salinity stability
3.6.3.3. Leukogram: total leukocytes (WBC)	cells/μL	Infection; toxicants; chronic stress; rough handling	Health/sanitary; Water quality; Animal; Transport; Human resources	**Pi 0** 20k−150k; **Pi 1** 150–200k or 15–20k; **Pi 2** >200k or < 15k. **Pop:** >20% Pi ≥ 1. **Sentinel:** ≥2 fish Pi = 2	Rule out toxic exposure; reduce handling/density; activate diagnostics (bacterial/viral); strengthen biosecurity and transport SOPs
3.6.3.4 Lymphocytes (%)	% of differential count	Chronic stress (cortisol); toxicants; viral disease; photoperiod disruption	Water quality; Health; Animal; Facilities/lighting; Transport	**Pi 0** 60–85%; **Pi 1** 50–59 or 86–95; **Pi 2** < 50 or >95. **Pop:** >20% Pi ≥ 1. **Sentinel:** ≥2 fish Pi = 2	Restore 12L:12D; reduce density/handling; check metals/pesticides; reinforce biosecurity; consider functional diets (β-glucans/probiotics)
3.6.3.5. Neutrophils (heterophils, %)	% of differential count	Acute bacterial infection; acute handling stress; tissue injury; myelotoxicants	Health; Animal; Transport; Water quality	**Pi 0** 10–30; **Pi 1** 30–40 or 5–10; **Pi 2** >40 or < 5. **Pop:** >20% Pi ≥ 1. **Sentinel:** ≥2 fish Pi = 2	Diagnostics + biosecurity; reduce air exposure/trauma; screen contaminants/mycotoxins if neutropenia; improve environment and recovery windows
3.6.3.6. Monocytes (%)	Chronic inflammation signal	Persistent infection; chronic stress; nutritional deficiency; incomplete treatments	Health; Therapeutics; Water quality; Nutrition	**Pi 0** 2–8; **Pi 1** 1–2 or 8–12; **Pi 2** < 1 or >12. **Pop:** >20% Pi ≥ 1. **Sentinel:** ≥2 fish Pi = 2	Review water quality and chronic infection; confirm complete targeted therapy; correct vitamins/trace minerals; reduce injury/handling
3.6.3.7. Eosinophils (%)	Parasite/hypersensitivity signal	Metazoan parasites; allergenic feeds; acute stress (↓)	Health; Water quality; Nutrition; Animal; Human resources	**Pi 0** 0–5; **Pi 1** 6–10 or 1–2; **Pi 2** >10 or < 1. **Pop:** >20% Pi ≥ 1. **Sentinel:** ≥2 fish Pi = 2	Skin/gill scrapes; targeted antiparasitic; density + water quality correction; review diet transitions; quarantine new batches
3.6.3.8. H/L ratio	Chronic stress index	Crowding; repeated handling; chronic NH_3_/${\text{NO}}_{2}^{-}$/hypoxia; photoperiod stress; toxicants	Animal; Water quality; Transport; Facilities/lighting; Human resources	**Pi 0** 0.15–0.50; **Pi 1** 0.51–0.70 (or < 0.15 if supplementation documented); **Pi 2** >0.70. **Pop:** >20% Pi ≥ 1. **Sentinel:** ≥2 fish Pi = 2	Reduce density; enforce recovery windows ≥ 48 h between procedures; correct water quality; normalize photoperiod; staff training + SOP consistency

Signal definition, main drivers (EPI), implicated management domains (DOM), operational triggers (Pi; population and sentinel criteria), and first-line corrective actions. Bold values indicate the operational thresholds and trigger criteria (Pi, population-based, and sentinel) used for welfare interpretation and decision-making in this guide.

**Table 5:** Internal welfare indicators in tilapia—core operational summary (IIC-Blood biochemistry indicators).

Indicator (IIC-Biochemistry)	Operational signal (what you measure/observe)	Main drivers (EPI keywords)	Main domains (DOM keywords)	Operational triggers (Pi/population/sentinel)	First-line corrective action (operational)
3.6.4.1. Glucose (mg/dl)	Acute stress/metabolic failure	Handling/air exposure; hypoxia; toxins; fasting/hepatic failure	Water quality; Animal; Transport; Human resources; Health	**Pi 0** 40–80; **Pi 1** 80–100 or 35–40; **Pi 2** >100 or < 35–40. **Pop:** >20% outside 40–80. **Sentinel:** ≥2 fish with severe extremes (e.g., >150 or < 30)	Reduce capture stress/air exposure; improve DO; correct toxins/leachates; manage fasting (24 h pre-transport); feeding correction if low; escalate to lactate/cortisol if persistent
3.6.4.2. Lactate (mmol/L)	Anaerobic load/acidosis risk	Severe hypoxia; intense pursuit; extreme density; blooms	Water quality; Animal; Human resources; Transport	**Pi 0** 1.5–5.0; **Pi 1** 5.0–6.0; **Pi 2** >6.0. **Pop:** >20% Pi ≥ 1. **Sentinel:** ≥2 fish Pi = 2	Immediate aeration/backup; reduce pursuit time; allow 24–48 h recovery; reduce density; control eutrophication/blooms; transport oxygenation/time control
3.6.4.3. Cortisol (ng/ml)	Primary endocrine stress axis	Photoperiod disruption; crowding; chronic hypoxia; rough handling; osmotic/thermal stress	Facilities/lighting; Animal; Water quality; Transport; Human resources	**Pi 0** 5–50; **Pi 1** 50–100 or 3–5; **Pi 2** >100 or < 3. **Pop:** >20% Pi ≥ 1. **Sentinel:** ≥2 fish with extremes (e.g., >150 or < 3)	Normalize 12L:12D; reduce density; DO >5 mg/L + continuous monitoring; gentle capture (air exposure < 3 min); salinity/temperature acclimation; review feeding protocols
3.6.4.4. Total proteins (TP, g/dl)	Nutrition/hepatic synthesis/immune stimulation	Chronic infection; dehydration; low-protein diets; hepatotoxins; fasting	Nutrition; Health; Water quality; Animal	Adults **Pi 0** 3.0–6.0; **Pi 1** 2.5–3.0 or 6.0–7.0; **Pi 2** < 2.5 or >7.0. Juveniles adjusted ranges as in text. **Pop:** ≥20% Pi ≥ 1. **Sentinel:** ≥2 fish Pi = 2	If high: diagnostics + quarantine + density/water correction. If low: reformulate protein quality; control aflatoxins/oxidized lipids; treat enteropathies; avoid prolonged fasting
3.6.4.5. Cholesterol (mg/dl)	Lipid metabolism/hepatic function	Plant diets, bile acid issues; oxidized oils; fasting; hepatopathy	Nutrition; Health; Water quality; Human resources	**Pi 0** 100–200; **Pi 1** 80–100 or 200–230; **Pi 2** < 80 or >230 (+ signs). **Pop:** ≥20% Pi ≥ 1. **Sentinel:** ≥2 fish Pi = 2	Adjust lipid/cholesterol/bile acids (plant diets); improve ingredient quality; reduce handling stress; if low: treat malabsorption/enteritis and reduce fasting
3.6.4.6. Triglycerides (mg/dl)	Energy balance/hepatic load	Overfeeding/hyperlipid diets; fasting/refeeding; thermal stress; hepatopathy	Nutrition; Water quality; Human resources; Health	Adults **Pi 0** 100–300; **Pi 1** 80–100 or 300–350; **Pi 2** < 80 or >350 (+ signs). Juveniles thresholds higher. **Pop:** ≥20% Pi ≥ 1. **Sentinel:** ≥2 fish Pi = 2	Balance energy:protein; adjust feeding to temperature; avoid abrupt fasting/refeeding; check water quality/density; evaluate hepatic health if persistent
3.6.4.7. Albumin (g/dl)	Hepatic synthesis/protein status	Protein deficiency; hepatotoxins; nephro/enteropathy; aflatoxins	Nutrition; Water quality; Health; Human resources	**Pi 0** 1.5–3.5; **Pi 1** 1.3–1.5 or 3.5–3.8; **Pi 2** < 1.3 or >3.8 (+ signs). **Pop:** ≥20% Pi ≥ 1; note stronger alert if ≥ 10% Pi = 2. **Sentinel:** ≥2 fish Pi = 2	Reformulate high-digestibility protein; control aflatoxins/oxidized oils/metals; treat enteropathies; correct water quality; review any hormone/therapeutics misuse
3.6.4.8. Globulins (g/dl)	Immune activation/immunosuppression	Chronic infection/parasitism; chronic stress; toxins; fasting	Health; Animal; Water quality; Nutrition	**Pi 0** 1.5–3.0; **Pi 1** 1.2–1.5 or 3.0–3.5; **Pi 2** < 1.2 or >3.5. **Pop:** ≥20% Pi ≥ 1; higher concern if ≥ 10% Pi = 2. **Sentinel:** ≥2 fish Pi = 2	If high: diagnostics + targeted treatment + biosecurity. If low: correct density/DO/nutrition; remove immunosuppressants; strengthen prevention/viral control
3.6.4.9. A/G ratio	Liver–immune balance	Hepatopathy; chronic infection; immunosuppression; low protein diets	Health; Nutrition; Water quality; Animal	**Pi 0** 0.8–1.5; **Pi 1** 0.7–0.79 or 1.5–1.8; **Pi 2** < 0.7 or >1.8. **Pop:** ≥20% Pi ≥ 1. **Sentinel:** ≥2 fish Pi = 2	Diagnostics (micro + hepatic); improve DO/NH_3_/${\text{NO}}_{2}^{-}$/pH; optimize protein/amino acids; apply targeted therapy; investigate feed/water hepatotoxins
3.6.4.10. Plasma ammonia (μmol/L)	Branchial excretion efficiency/NH_3_ load	High env. NH_3_; extreme density; gill dysfunction; hypoxia; blooms	Water quality; Animal; Nutrition; Health; Human resources	**Pi 0** < 100; **Pi 1** 100–200; **Pi 2** ≥200 or elevated + compatible signs. **Pop:** ≥20% Pi ≥ 1. **Sentinel:** ≥2 fish Pi = 2	Increase exchange/biofiltration; reduce organic load/overfeeding; correct DO; treat gill parasites if indicated; manage ammonia–salinity interaction; daily monitoring SOP
3.6.4.11. Electrolytes/osmolality (Na^+^/Cl^−^/K^+^; mOsm/kg)	Osmoregulatory integrity	Salinity shifts; dehydration; gill injury (NH_3_/${\text{NO}}_{2}^{-}$/parasites); hemodilution from sampling	Water quality; Animal; Health; Human resources; Transport	**Pi 0** within reference ranges; **Pi 1** 10–20% deviation in one parameter only; **Pi 2** >20% deviation or ≥2 parameters out of range and/or clinical signs. **Pop:** >20% Pi ≥ 1. **Sentinel:** ≥2 fish Pi = 2	Correct salinity gradually; verify intake water; control NH_3_/${\text{NO}}_{2}^{-}$; adjust density/depth; limit blood sampling volume/frequency; staff training on acclimation and sampling

Signal definition, main drivers (EPI), implicated management domains (DOM), operational triggers (Pi; population and sentinel criteria), and first-line corrective actions. Bold values indicate the operational thresholds and trigger criteria (Pi, population-based, and sentinel) used for welfare interpretation and decision-making in this guide.

**Table 6:** Internal welfare indicators in tilapia—laboratory-oriented module (IIC-Damage and Redox).

Indicator (IIC-Damage and Redox)	Operational signal	Main drivers (EPI keywords)	Main domains (DOM keywords)	Operational triggers (fold-change logic)	Corrective action (prioritized)
3.6.5.1. ALT/AST/LDH	Plasma enzymes reflecting hepatocellular/muscle injury or cytotoxicity	Cyanotoxins (microcystins); metals; leachates; oxidized oils; hypoxia; viral hepatopathy; traumatic handling	Water quality; Nutrition; Health; Animal; Human resources	**Pi 0:** ≤ 1.0 × reference/baseline. **Pi 1:** >1.0 × and < 2.0 × . **Pi 2:** ≥ 2.0 × . **Population:** alert if >25% Pi = 1; critical if ≥ 10% Pi = 2 or rising trend (2 samplings). **Sentinel AWE:** any fish Pi = 2 (as written in your text)	Remove suspected hepatotoxic source; verify feed peroxide/aflatoxins; improve DO; reduce density; antioxidant support; diagnostics for pathogens; enforce low-trauma handling SOPs
3.6.5.2. Malondialdehyde (MDA)	Lipid peroxidation (membrane oxidative damage)	Metals; leachates; hypoxia; thermal stress; high density; oxidized lipids; UV	Water quality; Nutrition; Animal; Human resources	**Pi 0:** within reference/baseline ( ≤ 1.0 × ). **Pi 1:** >1.0 × and < 2.0 × . **Pi 2:** ≥ 2.0 × . **Population:** alert >25% Pi = 1; critical ≥ 10% Pi = 2 or upward trend. **Sentinel AWE:** any fish Pi = 2	Eliminate contaminant source; prevent hypoxia (DO > 5); antioxidant supplementation (Vit E/C/Se as in text); reduce density; improve feed storage; shading/thermal buffering
3.6.5.3. SOD/CAT/GPx	Antioxidant defense activity (induction vs. exhaustion)	Moderate oxidative challenge (↑); salinity stress/ammonia–salinity interaction; cofactor deficiency; chronic severe stress (↓ exhaustion)	Water quality; Nutrition; Animal; Health	**Pi 0:** within reference/baseline ( ≤ 1.0 × ). **Pi 1:** deviation ≤ 1.8 × (up or down). **Pi 2:** ≥ 2.0 × change (marked induction or exhaustion). **Sentinel AWE:** any fish Pi = 2 OR “induction then collapse” pattern	Remove xenobiotics; correct salinity + NH_3_ simultaneously; restore mineral cofactors (Cu/Zn/Mn/Se) and antioxidants; improve aeration; reduce density; avoid concurrent stressors
3.6.5.4. Protein carbonyls	Irreversible protein oxidation (chronic cumulative damage)	Metals; xenobiotics; chronic hypoxia/thermal stress; oxidized oils; aging broodstock; high density/UV	Water quality; Nutrition; Animal; Genetics/reproduction	**Pi 0:** < 1.5 nmol/mg protein (or baseline). **Pi 1:** 1.5–2.5. **Pi 2:** >2.5. **Population:** alert >25% Pi = 1; critical ≥ 10% Pi = 2 or upward trend. **Sentinel AWE:** any fish Pi = 2 OR MDA + carbonyls rise together	Remove contaminants; correct DO/water quality; antioxidants and fresh oils; density reduction; shading; replace aged broodstock if relevant; strengthen monitoring of inputs

Tissue injury and oxidative stress markers with operational thresholds (fold-change approach) and corrective actions within EPI-DOM. Bold values indicate the operational thresholds and trigger criteria (Pi, population-based, and sentinel) used for welfare interpretation and decision-making in this guide.

#### 3.6.1 Respiratory rate (opercular ventilation)

Respiratory rate in tilapia, assessed via opercular ventilation (opercular beats per minute, OPM), is a highly sensitive internal indicator of environmental and handling-related changes (189, 190). This parameter directly reflects oxygen metabolic demand and gill-level gas-exchange efficiency, integrating respiratory function with energy metabolism and neuroendocrine regulation. Its modulation depends on factors such as temperature, body size, dissolved oxygen availability, and operational context (43, 191).

Under basal, controlled conditions, opercular ventilation in *Oreochromis niloticus* shows an inverse relationship with body mass and a direct relationship with temperature, a pattern widely described in tropical teleosts (189, 192). Body size also influences hypoxia tolerance, indirectly shaping ventilatory patterns (193). Under progressive hypoxia, a consistently described **biphasic** response emerges: an initial increase in opercular ventilation as a compensatory mechanism to sustain oxygen uptake, followed by a progressive decrease as stress intensifies and compensatory capacity becomes exhausted (191). This ventilatory decline is associated with systemic metabolic depression, reduced tissue ATP, and concomitant increases in plasma cortisol and glucose, indicating a shift from an adaptive phase toward physiological compromise (191).

Similarly, handling, confinement, and transport can induce acute increases in opercular ventilation even under normoxia, reflecting acute stress responses mediated by the neuroendocrine axis (43, 189). These respiratory changes correlate closely with physiological stress markers, consolidating respiratory rate as a reliable, rapid-response indicator under operational disturbances. Because the available literature does not establish universal reference values for opercular ventilation in tilapia—rather, it reports context-dependent ranges—this guide interprets respiratory rate as a **dynamic indicator**, always evaluated relative to the batch's population baseline rather than as a fixed absolute value. This approach helps distinguish adaptive physiological responses from functionally relevant alterations affecting welfare.

**EPI** **→** Acute or chronic hypoxia associated with reduced dissolved oxygen and aeration failures (191, 194, 195). Poor water quality, including ammonia and nitrite accumulation with impacts on gill function (196). Intensive handling (capture, confinement, transport) inducing acute respiratory stress responses (43, 189).

**DOM** **→** **Health and sanitary:** Opercular ventilation alterations reflect functional compromise of the respiratory system, particularly gill-level impairment linked to hypoxia, tissue damage, or sustained physiological stress. Respiratory rate acts as an early indicator of respiratory dysfunction before overt clinical signs appear (190, 191). **Animal (individual and population):** ventilatory response shows intra- and inter-individual variability related to body size, physiological status, and metabolic capacity; thus, it should be interpreted at the population level relative to a batch-specific baseline (189, 193). **Human resources** **+** **Transport and logistics:** capture, confinement, grading, and transport can acutely increase opercular ventilation even under normoxia, reflecting activation of the stress axis (43, 189). Respiratory rate allows estimation of the physiological load associated with handling.

**Individual scoring system (Pi)**

**Pi 0 (normal):** regular, rhythmic, symmetric opercular ventilation consistent with the system's baseline pattern, without surface gasping or evident changes in frequency or amplitude (191).

**Pi 1 (mild–moderate alteration):** detectable changes relative to baseline, including moderate tachypnea or bradypnea, increased opercular amplitude, irregular ventilation, or mild asynchrony. Compatible with an adaptive response to acute stress, incipient hypoxia, or handling, without severe respiratory compromise (189, 191).

**Pi 2 (severe alteration):** marked respiratory disturbance characterized by intense tachypnea or pronounced respiratory depression, shallow breathing, persistent surface gasping, or evident opercular asynchrony. Indicates exhaustion of compensatory mechanisms and severe respiratory compromise, associated with critical hypoxia or extreme physiological stress (191).

**Operational corrective action**

Immediately verify dissolved oxygen, temperature, ammonia, and nitrite. Correct aeration and water-exchange failures and reduce organic loading when values are outside the system's operational range. Reduce stocking density/biomass when feasible. Temporarily suspend handling procedures (grading, biometrics, sampling, and harvest) until normalization of the respiratory pattern. Adjust hydraulic flows, discharge rates, and currents in RAS, BFT, or ponds to avoid ventilatory overexertion; identify dead zones, thermal stratification, or poorly oxygenated areas. During transport, review loading density, oxygenation, and thermal stability; avoid overloading and prolonged transfer times. Train personnel to recognize early tachypnea, bradypnea, surface gasping, and opercular asynchrony, and establish clear stop-criteria when **Pi** **≥** **1**. Reassess respiratory status every 12–24 h until normalization. If **Pi** **≥2** persists, escalate to metabolic indicators (glucose, lactate, and cortisol) and conduct a system-wide review.

#### 3.6.2 Heart rate

Heart rate is a key internal indicator of physiological state in tilapia because it reflects functional integration between the cardiovascular system, energy metabolism, and autonomic regulation. Unlike respiratory rate, its assessment requires instrumental techniques—such as Doppler, electrocardiography, or implantable sensors—limiting routine use primarily to laboratory or research contexts. In teleost fish, heart rate may increase or decrease depending on the type, intensity, and duration of the stressor. During the initial phase of acute stress, sympathetic activation often induces transient tachycardia; however, under prolonged hypoxia or severe stress, **adaptive bradycardia** is frequently observed, associated with metabolic depression and energy conservation (191). In Tilapia, this response has been described as part of a physiological strategy to maintain the balance between cardiac ATP supply and demand.

Experimental studies show that *Oreochromis niloticus* can display high cardiovascular tolerance to hypoxia and even transient anoxia, maintaining relatively stable cardiac output through adjustments in stroke volume and blood-flow redistribution (197, 198). Nevertheless, when compensatory mechanisms are exceeded, profound bradycardia and high rhythm variability may precede physiological collapse. In this context, heart rate should be interpreted as a confirmatory indicator—useful for validating alterations detected through external, respiratory, or biochemical indicators—and for distinguishing reversible adaptive responses from physiological depression consistent with sustained physiological distress.

Ammonia toxicity is a relevant stressor in tilapia with direct effects on cardiovascular function. Prolonged exposure to sublethal concentrations of unionized ammonia can induce significant reductions in heart rate and increased rhythm variability, accompanied by metabolic depression, even when brief acute exposures may be tolerated without immediate changes (199).

**EPI** **→** Prolonged or severe hypoxia associated with bradycardia and metabolic depression (191, 197). Ammonia toxicity with reduced heart rate and increased rhythm variability (199). Handling, transport, or confinement stress potentially inducing transient tachycardia (physiologically grounded inference supported by hypoxia/stress-response patterns described in 191). Anesthesia/sedation, particularly with poorly adjusted dosing, with direct effects on cardiovascular control (200).

**DOM** **→** **Animal:** cardiac response as a confirmatory indicator of physiological state under hypoxia/anoxia (191, 197, 198). **Health and sanitary:** systemic physiological compromise under hypoxia and associated metabolic depression (191). **Therapeutics:** sedatives/anesthetics can alter heart rate and must be explicitly considered when interpreting results (200). **Transport and logistics:** transfers may precipitate hypoxia/stress; thus, heart rate supports validation of transport-related physiological load (191). **Natural resources (water -environment domain-):** hypoxia as a primary driver of bradycardia/cardiovascular adjustment and ammonia as a stressor reducing heart rate and increasing variability (191, 197–199). **Human resources:** requirement for instrumental measurement (Doppler/ECG) and standardized measurement protocols.

**Individual scoring system (Pi)**

**Pi 0 (normal):** stable, regular heart rate consistent with the baseline pattern under controlled conditions. *Note:* there is no universal basal value for tilapia; assessment must be relative to the baseline of the experimental system or batch.

**Pi 1 (mild–moderate alteration):** moderate tachycardia or bradycardia relative to baseline, without marked arrhythmia. Compatible with transient stress or a reversible adaptive response (191, 199).

**Pi 2 (severe alteration):** marked and sustained tachycardia or bradycardia accompanied by high rhythm variability or clear irregularity, indicating significant cardiovascular compromise and physiological depression (197, 199).

**Operational corrective action**

Correct hypoxia and/or nitrogenous toxicity in water immediately before continuing any procedure; optimize aeration and water exchange as first-line actions. Minimize handling and invasive procedures and postpone non-essential sampling until cardiovascular stability is restored. Review and adjust sedation/anesthesia protocols; avoid overdosing and ensure continuous physiological monitoring during experimental procedures. For transport, reduce transfer time, control loading density and oxygenation, and avoid moving fish during detected cardiovascular stress. Ensure that only trained personnel perform cardiac measurements (Doppler/ECG) and standardize baseline conditions and measurement time points to avoid misinterpretation. Reassess after environmental correction; if **Pi** **=** **2** persists, integrate metabolic assessment (lactate, glucose) and consider isolating the affected batch while conducting a broader system review.

#### 3.6.3 Hematological indicators

Hematological indicators are a central pillar in evaluating **physiological welfare** in tilapia because they sensitively reflect the interaction between **immune status**, **oxygen-transport capacity**, and responses to environmental, nutritional, and handling stressors. Parameters such as hematocrit, hemoglobin, total and differential leukocyte counts (lymphocytes, neutrophils, monocytes, and eosinophils), and the H/L ratio enable early identification of hypoxia, anemia, acute or chronic inflammation, immunosuppression, and exposure to contaminants. When interpreted integratively within the EPI-DOM framework, hematological alterations can be linked to specific failures across management domains—including water quality, biosecurity, animal handling, nutrition, and production pressure—thereby supporting evidence-based operational corrective actions. Collectively, blood parameters function as **sentinels of internal welfare**, complementing external and behavioral assessment and providing an objective readout of the cumulative impact of stressors in aquaculture systems. Below, key internal hematological indicators in tilapia are described (operational ranges for juveniles–adults).

##### 3.6.3.1 Hematocrit (Hct)

The proportion of erythrocytes relative to total blood volume; reflects oxygen-carrying capacity, hydration status, osmoregulation, and erythropoietic function (201, 202).

**Physiological ranges:** 26.17%−33.19% (203) and 25%−45% in healthy adult tilapia (201, 204). Disease outbreaks may increase Hct to 37.2%−58.9% (205).

**EPI** **→**

**Hct** **↓** **(low**, ** < 25%):** anemia due to repeated bleeding (206). Methylmercury toxicity → normocytic normochromic anemia (207). Nutritional deficiencies and feed restrictions (208). Viral infections such as TiLV (166).

**Hct** **↑** **(high**, **>45%):** exposure to organochlorine pesticides (209). Osmotic stress due to abrupt salinity changes (210). Dehydration associated with high stocking density and poor water quality (211). Additionally influenced by hypoxia, infection, temperature, nutritional stress (166, 212, 213), and toxins/contaminants (214–216).

**DOM** **→ Natural resources (water)** **+** **Health/sanitary:** monitoring of heavy metals, filtration, water treatment, and reduction of contamination (207, 209). **Feeding and nutrition** **+** **Economic and productive:** diets containing iron (60–150 mg/kg), vitamin B12, folic acid, and copper; avoid decreases in nutritional quality (204). **Animal** **+** **Human resources:** gradual salinity acclimation (2–4 ppt/day), training in osmotic management, and adjustment of stocking densities (210, 211).

**Individual scoring system (Pi)**

**0**: Hct within the physiological range (25%−45%).

**1**: moderate deviation: Low → 20–24%, High → 46–50%. Compatible with mild–moderate stress or early-stage impairment.

**2**: severe alteration: Low → < 20%, High → >50%. Compatible with significant anemia, intoxication, severe dehydration, or osmotic failure.

**Population-based criterion**

If **>20%** of sampled fish have **Pi** **≥** **1**, a population-level hematological alteration is considered present and intervention is required.

**Sentinel AWE criterion**

If the sample includes **≥2 fish with Pi** **=** **2**, an AWE is considered present, regardless of pond size.

**Operational corrective action**

Review water quality (ammonia, nitrite, oxygen, and heavy metals). Verify salinity and implement gradual adjustments (2–4 ppt/day; 210, 211). Review stocking density and reduce if needed. Evaluate the nutritional plan and supplement iron, B12, folic acid, and copper. Review health history and rule out infection-related anemia (viral/bacterial). Train personnel in sampling techniques, osmotic management, and low-stress procedures.

##### 3.6.3.2 Hemoglobin (Hb)

Concentration of the oxygen-transport protein in blood. A direct indicator of oxygenation capacity, oxidative stress, and heavy-metal toxicity (204, 217).

**Operational range in adult tilapia:** 7–12 g/dl (201, 204).

**EPI** **→**

**Hb** **↓** **(reduced**, ** < 7 g/dl):** methylmercury intoxication (207). Iron/B12/folate deficiencies (204). Toxicant-induced hemolysis (217). Repeated blood loss (206).

**Hb** **↑** **(elevated**, **>12 g/dl):** organochlorine pesticides (209). Chronic hypoxia due to high stocking density or inadequate aeration (204). Osmotic stress due to abrupt salinity changes (210).

**DOM** **→** **Natural resources (water):** chemical contamination, pesticides, heavy metals (207, 209). **Feeding and nutrition:** diets deficient in iron/B12/folates (204). **Animal (population)** **+** **Human resources:** high stocking densities, hypoxia, handling that causes hemolysis or blood loss, poor sampling technique, and inadequate osmotic management (204, 210). **Economic and productive:** cost-cutting in diets or aeration that compromises Hb (204).

**Individual scoring system (Pi)**

**0**: 7–12 g/dl

**1**: 6–6.9 or 12.1–14 g/dl

**2**: < 6 or >14 g/dl

**Population-based criterion**

If **>20%** of sampled fish have **Pi** **≥** **1**, population-level hematological deterioration is considered present and intervention is required.

**Sentinel AWE criterion**

If the sample includes **≥2 fish with Pi** **=** **2**, an AWE is considered present regardless of overall batch percentage.

**Operational corrective action**

**For** **↓** **Hb:** assess heavy-metal contamination, adjust diets with iron and B12, strengthen biosecurity and water quality management.

**For** **↑** **Hb:** evaluate chronic hypoxia and stocking density, increase aeration, and investigate potential exposure to organochlorine pesticides.

##### 3.6.3.3 Leukogram: total leukocytes (WBC)

Total leukocyte count reflects immune status and systemic stress.

**Operational range:** 20,000–150,000 cells/μl (201, 204). WBC responds to infections, toxicants, handling, and water-quality challenges (218).

**EPI** **→**

**↑Leukocytosis (>150,000/μl):** organochlorine pesticides can induce systemic inflammation (209). Bacterial infections (*Aeromonas, Streptococcus*) associated with poor biosecurity (219, 220). Rough handling, frequent capture, and stressful transport (219).

**↓Leukopenia (< 20,000/μl):** myelotoxic heavy metals (methylmercury) (207). Severe chronic stress due to excessive stocking density or persistently poor water quality (211). Viral diseases in the absence of prevention and quarantine programs (204).

**DOM** **→** **Natural resources (water):** chemical residues, pesticides, and heavy metals in culture water (207, 209). **Health and sanitary:** biosecurity failures and lack of robust prevention/control programs for pathogens (204, 220). **Animal:** high densities, chronic stress, and rough handling promoting inflammation and immune dysfunction (211, 219). **Transport and logistics:** transfers without welfare SOPs, with stressful handling that contributes to leukocytosis and systemic inflammation (219). **Human resources:** need for training in biosecurity, gentle handling, and operational stress reduction (204, 219). **Economic and productive:** production pressure maintaining high densities and compromising welfare and immunocompetence (211).

**Individual scoring system (Pi)**

**0:** WBC within 20,000–150,000 cells/μl.

**1 (moderate deviation):**

Mild leukocytosis: 150,000–200,000
Mild leukopenia: 15,000–20,000

**2 (severe deviation):**

200,000 (severe systemic inflammation/pathogens/toxicants)
< 15,000 (severe immunosuppression)

**Population-based criterion**

If **>20%** of sampled fish have **Pi** **≥** **1**, population-level immune dysfunction is considered present.

**Sentinel AWE criterion**

If the sample includes **≥2 fish with Pi** **=** **2**, classify as an AWE regardless of pond size or overall prevalence.

**Operational corrective action**

Review water quality and rule out toxic exposure. Reduce density and the frequency/intensity of handling. Activate strict biosecurity protocols. Improve capture, grading, and transport procedures. Assess the presence of bacterial or viral pathogens.

##### 3.6.3.4 Lymphocytes

Lymphocytes are the main fraction of the differential leukogram and reflect adaptive immune competence (204, 221).

**Physiological range:** 60%−85% of total leukocytes (201, 204).

**EPI** **→**

**↑Lymphocytosis (>85%):** exposure to organochlorine contaminants → abnormal immune stimulation (209). Altered photoperiods (6L:6D) → increased lymphocyte proportion (221). Chronic immune stimulation due to sustained environmental antigenic load (204).

**↓Lymphopenia (< 60%):** methylmercury → severe lymphopenia (207). Chronic stress with elevated cortisol due to handling, density, and transport (219, 221). Immunosuppression due to toxicants or viral disease (204, 217).

**DOM** **→** **Natural resources (water):** organochlorines and xenobiotics associated with lymphocytosis; heavy metals such as mercury associated with lymphopenia (207, 209). **Health and sanitary:** high antigenic load, opportunistic pathogens, viral immunosuppression (204, 217). **Animal** **+** **Human resources:** chronic stress from high densities and frequent handling → cortisol-mediated lymphopenia (219, 221). **Transport and logistics:** transport without SOPs increases cortisol and depresses lymphocytes (219). **Human resources** **+** **Animal (population):** poor photoperiod management → lymphocytosis via circadian disruption (221). **Economic and productive:** production pressure sustaining high densities and deteriorating water quality, increasing immunosuppression risk (211).

**Individual scoring system (Pi)**

**0:** 60%−85% (within physiological range).

**1:** 50%−59% (mild lymphopenia) or 86–95% (moderate lymphocytosis). Compatible with subclinical stress, environmental change, or mild irritation.

**2:** < 50% (severe lymphopenia) or >95% (marked lymphocytosis). Compatible with immunosuppression, intoxication, viral disease, or contaminant exposure.

**Population-based criterion**

If **>20%** of sampled fish have **Pi** **≥** **1**, population-level immune alteration is present and immediate intervention is recommended.

**Sentinel AWE criterion**

If **≥2** fish in the sample have **Pi** **=** **2**, classify as an AWE regardless of pond size.

**Operational corrective action**

Review water quality to rule out pesticides/organohalogen compounds and heavy metals. Adjust photoperiod to 12L:12D using automatic timers. Reduce density and minimize handling. Strengthen biosecurity (biological filtration, reduction of organic loading, pathogen control). Implement dietary immunostimulants (e.g., β-glucans, probiotics). Review transport history and improve low-stress capture protocols. Train personnel in welfare, basic immunology, and lighting management.

##### 3.6.3.5 Neutrophils (heterophils)

Neutrophils (heterophils) are key cells of innate defense via phagocytosis and constitute a first-line response to infection and tissue damage (204, 219).

**Physiological range:** 10%−30% of the differential leukogram in healthy tilapia (201, 204).

**EPI** **→**

**↑Neutrophilia (>30%):** acute bacterial infections (*Aeromonas, Streptococcus*) → systemic inflammation (219, 220). Acute stress from handling/transport, air exposure, rough capture, prolonged transfer (219). Tissue injury from abrasion, biting, or collisions (204).

**↓Neutropenia (< 10%):** methylmercury exposure → severe granulocytopenia (207, 217). Myelotoxicants (pesticides, mycotoxins) (209). Severe chronic stress due to extreme densities or persistently poor water quality (211).

**DOM** **→** **Health and sanitary:** biosecurity failures and lack of pathogen control (219, 220). **Animal:** rough handling, air exposure, aggressive grading (219). **Transport and logistics:** prolonged transfers without SOPs, insufficient oxygenation (219). **Natural resources (water):** pesticide/heavy-metal contamination (207, 209, 217). **Economic and productive:** production pressure maintaining high densities (211).

**Individual scoring system (Pi)**

**0:** 10%−30% (physiological range).

**1:** 30%−40% or 5%−10%. Compatible with mild infection, acute stress, or early immunosuppression.

**2:** >40 or < 5%. Compatible with severe acute infection, intoxication, marked immunosuppression, or extremely stressful handling.

**Population-based criterion**

If **>20%** of sampled fish have **Pi** **≥** **1**, a population-level hematological alteration is present.

**Sentinel AWE criterion**

If the sample includes **≥2 fish with Pi** **=** **2**, classify as an AWE regardless of pond size.

**Operational corrective action**

Review water quality (ammonia, nitrite, contaminants, pesticides, heavy metals). Reduce handling and air exposure; implement low-trauma SOPs for capture and transport. Strengthen biosecurity with bacterial diagnostics and density control. For neutropenia: investigate heavy metals or mycotoxins; adjust diet with immunostimulants (β-glucans, probiotics); improve environment and reduce chronic stress. Train staff in low-stress techniques and sanitary management.

##### 3.6.3.6 Monocytes

Phagocytic cells that are macrophage precursors; involved in chronic inflammation, antigen presentation, and clearance of damaged tissues (204).

**Operational range:** 2%−8% of the differential count (201, 204).

**EPI** **→**

**↑Monocytosis (>8%):** chronic bacterial infections, granulomatous inflammation, phagocytosis of necrotic tissue, and dietary supplementation with immunostimulants (209, 222).

**↓Monocytopenia (< 2%):** immunosuppression due to toxicants and/or nutritional deficiencies (204).

**DOM** **→ Health and sanitary** **+** **Therapeutics** **+** **Animal** **+** **Natural resources (water):** unresolved persistent infections, inadequate/incomplete antibiotic treatments, poor water quality, and handling-related injuries (204). **Feeding and nutrition:** dietary immunosuppressors (mycotoxins, heavy metals) and diets deficient in vitamins A, C, E, and selenium (204).

**Individual scoring system (Pi)**

**0:** 2%−8% (physiological range).

**1:** 1%−2% or 8%−12% (moderate deviation).

**2:** < 1 or >12% (severe chronic inflammation or marked immunosuppression).

**Population-based criterion**

If **>20%** of fish have **Pi** **≥** **1**, a population-level hematological alteration is present.

**Sentinel AWE criterion**

If the sample includes **≥2 fish with Pi** **=** **2**, classify as an AWE.

**Operational corrective action**

Review water quality (ammonia, nitrite, and organic loading). Improve biosecurity and ensure complete antimicrobial treatments when indicated. Implement UV or ozone disinfection as appropriate. Correct nutritional deficiencies (vitamins A, C, E, and Se). Reduce handling-related injuries and provide appropriate shading. Eliminate sources of mycotoxins or heavy metals.

##### 3.6.3.7 Eosinophils

Eosinophils are granulocytes involved in immune responses to metazoan parasites and hypersensitivity processes. Their proportion is typically low in healthy fish; they increase during parasite infestations and decrease with acute stress or immunosuppression (204).

**Physiological range:** 0%−5% of the differential leukogram (201, 204).

**EPI** **→**

**↑Eosinophilia (>5%):** parasitic infestations (monogeneans, protozoa, ectoparasitic crustaceans) and poor water quality favoring parasite proliferation (204); potential hypersensitivity reactions to certain dietary ingredients (204).

**↓Eosinopenia (< 1%):** acute stress with cortisol elevation due to handling or transport (219); immunosuppression associated with corticosteroids (204).

**DOM** **→** **Health and sanitary:** parasitological surveillance, outbreak control, targeted treatments (204). **Animal** **+** **Human resources:** density, capture/grading stress, rough handling (204, 219). **Natural resources (water):** water quality modulating parasitism risk (204). **Feeding and nutrition:** assessment of allergenic ingredients and diet transitions (204). **Transport and logistics:** minimize transfer-related stress (219). **Human resources:** training in gentle handling and parasite-management SOPs (219).

**Individual scoring system (Pi)**

**0:** 0%−5% (physiological range).

**1:** moderate eosinophilia (6%−10%) or mild eosinopenia (1%−2%).

**2:** eosinophilia >10% or eosinopenia < 1% (significant parasitosis/hypersensitivity or severe immunosuppression).

**Population-based criterion**

If **>20%** of sampled fish have **Pi** **≥** **1**, population-level immune alteration associated with parasitism or stress is present.

**Sentinel AWE criterion**

If the sample includes **≥2 fish with Pi** **=** **2**, classify as an AWE regardless of pond size.

**Operational corrective action**

Initiate parasitological diagnosis (skin/gill scrapes). Apply formalin or hydrogen peroxide treatments as indicated by diagnosis. Reduce density and improve water quality (ammonia, nitrite, oxygen). Review diets for potential allergens and adjust formulations. Train personnel in gentle capture and low-stress handling. Implement strict quarantine for new batches (14–21 days).

##### 3.6.3.8 H/L ratio

The heterophil/lymphocyte ratio (H/L) is a robust biomarker of chronic stress in teleosts. Under prolonged stress, elevated cortisol increases neutrophils and decreases lymphocytes, raising the H/L ratio. It is an integrated indicator of welfare and immune stress and is often more stable than cortisol alone (219, 221).

**Operational range:** 0.15–0.50 in healthy tilapia under optimal conditions (219, 223).

**EPI** **→**

**H/L elevated (>0.50):** chronic stress due to crowding (211, 219). Repeated handling, frequent capture, transport without SOPs (219). Chronic poor water quality with ammonia/nitrite and low oxygen (224). Altered photoperiods (6L:6D) increase H/L (221). Prolonged exposure to environmental toxicants (209). Chronic thermal stress outside the optimal range (204).

**H/L decreased (< 0.15):** optimal welfare/low stress conditions (219) and/or dietary immunostimulation (β-glucans, probiotics) (222).

**DOM** **→** **Animal:** density, capture, manipulation frequency, grading (211, 219). **Economic and productive:** production pressure increasing density or limiting investment in monitoring (209, 211). **Human resources:** insufficient training in low-stress handling and environmental monitoring (219). **Natural resources (water -environment domain-):** ammonia, nitrite, oxygen, contaminants, chronic thermal variability (204, 224). **Photoperiod/temperature/solar radiation:** circadian disruption factors (221). **Transport and logistics:** transfers without protocols, repeated captures (219). **Health and sanitary:** persistent exposure to environmental toxicants (209). **Feeding and nutrition:** use of functional ingredients and immunostimulants (222).

**Individual scoring system (Pi)**

**0:** 0.15–0.50 (physiological range; absence of chronic stress).

**1:** 0.51–0.70 or < 0.15 with documented supplementation. Compatible with moderate stress or early dysfunction.

**2:** >0.70. Compatible with severe chronic stress (crowding, poor water quality, altered photoperiod, toxicants).

**Population-based criterion**

If **>20%** of sampled fish have **Pi** **≥** **1**, population-level chronic stress is present, consistent with persistent failures in management and/or environment.

**Sentinel AWE criterion**

If **≥2** fish present **Pi** **=** **2**, classify as an AWE regardless of pond size.

**Operational corrective action**

Reduce densities to system-appropriate optimal levels. Minimize repeated handling; implement gentle capture and consider anesthetics when appropriate. Establish ≥48 h recovery periods between procedures. Correct water quality and perform water exchanges as needed. If relevant, adjust photoperiod to 12L:12D using timers. Implement periodic contaminant screening. Mitigate chronic thermal stress (e.g., shading, thermal buffering where feasible). Train staff in early stress recognition and consistent welfare monitoring.

#### 3.6.4 Blood biochemistry indicators

Blood biochemistry is one of the core pillars of internal welfare assessment in Tilapia because it directly reflects the organism's physiological, metabolic, and immunological status. Plasma indicators—glucose, lactate, cortisol, total proteins, cholesterol, triglycerides, albumin, globulins, the A/G ratio, plasma ammonia, and electrolytes—enable early detection of homeostatic disruption derived from failures in management, environment, or interaction, in accordance with the EPI-DOM framework. Their integrated interpretation supports recognition of nutritional imbalances, hepatic dysfunction, inflammatory states, immunosuppression, and metabolic stress. Each parameter is presented in the table with its operational description, expected physiological values (operational range for juveniles–adults), associated risk factors, implicated management domains, individual scoring (Pi), population criterion, sentinel AWE threshold, and the corresponding operational corrective action. This standardized approach enables immediate decision-making and supports continuous welfare improvement programs in both laboratory and commercial aquaculture systems.

##### 3.6.4.1 Glucose

Plasma glucose is the classical biomarker of the **secondary response to acute stress**. Cortisol and catecholamines induce glycogenolysis and gluconeogenesis, increasing glucose following handling, transport, hypoxia, or toxin exposure (219, 225).

**Operational range:** 40–80 mg/dl in resting tilapia (204, 219).

**EPI** **→**

**↑** **Hyperglycemia (>80 mg/dl):** Acute stress (219, 225). Hypoxia, toxins (218).

**↓Hypoglycemia (< 40 mg/dl):** Pesticides (209). Fasting, hepatic failure (218).

Environment: pesticides (209), toxic leachates (218), hypoxia and thermal variability (204). Management practices: air exposure during capture (219), transport without fasting or acclimation (219), high stocking densities (211). Human–animal interaction: rough handling, untrained staff, absence of welfare SOPs (20, 225).

**DOM** **→** **Natural resources (water):** contaminants, pesticides, and leachates (209, 218). **Health and sanitary:** failures to control contaminants and environmental biosecurity (218). **Animal:** rough handling, inadequate aeration, poor acclimation (219). **Transport and logistics:** absent fasting, excessive densities, thermal variation (219). **Human resources:** insufficient training in stress reduction and control of operational stressors (225). **Economic and productive:** production pressure sustaining high densities (211).

**Individual scoring system (Pi)**

**0:** 40–80 mg/dl (physiological range without stress).

**1:** 80–100 or 35–40 mg/dl (moderate, reversible response).

**2:** >100 or < 35–40 mg/dl (acute stress or metabolic failure).

**Population criterion**

If **>20%** of sampled fish show values outside the normal range (40–80 mg/dl), this is considered a population-level alteration of glycemic metabolism associated with acute or chronic stress and requires operational intervention (any deviation counts as **Pi** **≥** **1**).

**Sentinel AWE criterion**

If **≥2 fish** in the sample present **Pi** **=** **2**, i.e., **severe hyperglycemia (>150 mg/dl)** or **marked hypoglycemia (< 30 mg/dl)**, this constitutes an **AWE**, regardless of lot size.

**Operational corrective action**

**Hyperglycemia:** reduce air exposure to < 3 min; apply gentle capture; use anesthetics/sedatives; fast 24 h before transport; keep density appropriate to biomass; thermal acclimation at ~2 °C/h. Eliminate leachate sources; install filtration; monitor emerging toxins. Maintain DO >5 mg/L and system-appropriate densities.

**Hypoglycemia:** monitor and eliminate pesticide exposure; feed at least 2–3 times/day (more if appropriate to life stage); eliminate hepatotoxins; consider dietary hepatoprotectants.

##### 3.6.4.2 Lactate

Plasma lactate reflects anaerobic metabolism when ATP demand exceeds aerobic capacity (hypoxia, intense activity, severe stress). It is a sensitive biomarker of metabolic stress and possible acidosis (226, 227).

**Operational range:** 1.5–5.0 mmol/L at rest; in GIFT tilapia: 4.61 mmol/L (day 0), 4.78 mmol/L (day 20), 4.54 mmol/L (day 40) (227). **Note:** < 1.5 mmol/L may reflect ractopamine exposure (227) or improved aerobic metabolism.

**EPI** **→**

**↑** **Hyperlactatemia (>5 mmol/L):** severe hypoxia, toxic blooms, pursuit during capture, or extreme density (226).

Environment: cyanobacterial blooms, eutrophication, severe hypoxia (226). Management practices: pursuit during capture, inadequate aeration, excessive density in ponds or transport (219). Human–fish interaction: rough handling, prolonged capture time, inadequate techniques, failures in operational monitoring (20, 219).

**DOM** **→** **Natural resources (water -environment domain-):** toxic blooms, hypoxia, nutrient overload (226). **Health and sanitary:** lack of algal/eutrophication control. **Animal** **+** **Human resources:** pursuit, poor capture, extreme densities (219), insufficient training in gentle handling and preventive aeration (20, 219). **Transport and logistics:** overloading, prolonged trips without adequate oxygenation (219). **Economic and productive:** production pressure favoring high densities and insufficient oxygenation capacity.

**Individual scoring system (Pi)**

**0:** 1.5–5.0 mmol/L (physiological range).

**1:** 5.0–6.0 mmol/L (moderate, reversible alteration).

**2:** >6.0 mmol/L (severe metabolic stress/acidosis risk). **Note:** values < 1.5 mmol/L are not inherently adverse if other indicators remain normal (efficient aerobic metabolism).

**Population criterion**

If **>20%** of sampled fish show **Pi** **≥** **1**, a population-level lactate metabolism problem is assumed and requires intervention.

**Sentinel AWE criterion**

If **≥2 fish** show **Pi** **=** **2**, this constitutes an **AWE** due to severe metabolic alteration.

**Operational corrective action**

Under hyperlactatemia: reduce nutrient loading and control eutrophication; immediate aeration and DO >5 mg/L with backup systems; reduce density and organic load. Minimize pursuit (efficient capture, appropriate nets) and allow 24–48 h recovery. Keep transport short and adequately oxygenated (bags) or provide continuous oxygenation.

##### 3.6.4.3 Cortisol

Cortisol is the primary stress corticosteroid in teleosts, mediating endocrine responses to stressors through regulation of energy metabolism, osmoregulation, and immune function (219, 221).

**Operational range:** 5–50 ng/ml under basal conditions (219, 221).

**Abnormalities:**

**Hypercortisolemia:** >100 ng/ml
**Hypocortisolemia:** < 5 ng/ml

**EPI** **→**

**↑Hypercortisolemia (>100 ng/ml):** altered photoperiod (6L:6D) disrupting the HPI axis (221); high density and production pressure (211, 219); inappropriate feeding rates (225); abrupt salinity changes causing osmotic stress (210); rough handling, transport, air exposure (219); chronic hypoxia (< 4 mg/L) (204); social stress (hierarchies, aggression) (204).

**↓** **Hypocortisolemia (< 5 ng/ml):** HPI axis exhaustion due to severe chronic stress (204); dietary adaptogens/stress modulators (221).

- Also consistent with optimal welfare/low stress (219).

**DOM** **→** **Natural resources (water -environment domain-):** hypoxia, thermal or salinity changes (204, 210). **Animal:** density, hierarchies, capture, handling (204, 219). **Facilities/lighting management (environmental domain):** unstable lighting (221). **Transport and logistics:** rough handling, air exposure (219). **Feeding and nutrition:** inadequate feeding rates (225). **Human resources:** training deficits are implicated across drivers. **Economic and productivity:** excessive biomass due to production pressure (211).

**Individual scoring system (Pi)**

**0:** 5–50 ng/ml.

**1:** moderately out of range: 50–100 or 3–5 ng/ml.

**2:** critical values: >100 ng/ml (severe hypercortisolemia) or < 3 ng/ml (marked hypocortisolemia).

**Population criterion**

If **>20%** of sampled fish show **Pi** **≥** **1**, this indicates population-level alteration of the HPI axis and requires immediate intervention.

**Sentinel AWE criterion**

If **≥2 fish** show **Pi** **=** **2**, i.e., **cortisol** **>150 ng/ml** or ** < 3 ng/ml**, this constitutes an **AWE**, regardless of lot size.

**Operational corrective action**

Normalize photoperiod (12L:12D) (221). Reduce density; improve aeration (DO >5 mg/L); strengthen 24/7 monitoring. Implement gentle capture and minimize time out of water (< 3 min) (228). Acclimate salinity (2–4 ppt/day) and temperature ( ≤ 2 °C/h). Review feeding protocols (rate, frequency, quality). Mitigate social stress (size grading, refuges). Train staff in welfare SOPs.

##### 3.6.4.4 Total proteins (TP)

Total plasma proteins (TP) represent the sum of albumin and globulins and reflect protein nutritional status, hepatic synthetic capacity, colloid osmotic balance, and immune activation (201, 229). They are essential for oncotic pressure, molecular transport, and immune modulation. Changes in TP indicate nutritional, hepatic, renal, or immunological disturbances (204).

**Operational range in tilapia:** 3.0–6.0 g/dl (30–60 g/L) in healthy adults (201, 204, 229). Juveniles may present 2.5–5.0 g/dl (230).

**EPI** **→**

**↑** **Hyperproteinemia (>6.0 g/dl):** chronic immune stimulation due to bacterial/parasitic infections increasing globulins (231); dehydration associated with excessive density or poor water quality (232); supplementation with immunostimulants (BergaPur^®^, β-glucans) increasing globulins (222); winter stress with increased pathogen susceptibility (231).

**↓Hypoproteinemia (< 3.0 g/dl):** protein-deficient or low-quality diets (< 25% CP) (232, 233); hepatic dysfunction due to aflatoxins, oxidized lipids, heavy metals, or steroid use (234); enteropathies or nephropathies with protein loss (206); intestinal parasitosis with malabsorption; prolonged fasting >7 days (235).

**DOM** **→** **Feeding and nutrition:** protein level/quality, digestibility, amino acid balance (232, 233). **Health and sanitary:** chronic infections, parasitosis, hepatotoxins, nephropathies, enteritis (231, 234). **Natural resources (water -environment domain-):** ammonia, nitrite, CO_2_, heavy metals, overall water quality (206). **Animal:** density, prevention of seasonal stress, nutrition adjusted to temperature (231). **Therapeutics:** management of intoxications, mycotoxin binders, enteropathy control. **Human resources:** training in formulation, storage, feeding, and disease diagnosis. **Economic and productive:** pressure to reduce dietary protein or sustain excessive densities (232).

**Individual scoring system (Pi)—adults**

**0 (normal):** 3.0–6.0 g/dl.

**1 (mild alteration):** 2.5–3.0 or 6.0–7.0 g/dl without clinical signs.

**2 (moderate–severe alteration)**: < 2.5 or >7.0 g/dl, or accompanied by compatible clinical signs (lethargy, ascites, pale or darkened gills, weight loss, edema).

**Individual scoring system (Pi)—juveniles**

**0 (normal):** 2.5–5.0 g/dl.

**1 (mild alteration):** 2.0–2.5 or 5.0–6.0 g/dl without clinical signs.

**2 (moderate–severe alteration):** < 2.0 or >6.0 g/dl, or accompanied by compatible clinical signs (lethargy, ascites, pale or darkened gills, weight loss, edema).

**Population criterion**

If **≥20%** of the lot has **Pi** **≥** **1**, this indicates population-level deterioration of protein nutrition/hepatic status or chronic infection and requires intervention.

**Sentinel AWE criterion**

If **≥2 fish** show **Pi** **=** **2** in the same lot, this suggests an **AWE** related to hepatic, nutritional, or infectious failure requiring immediate correction.

**Operational corrective action**

**Hyperproteinemia:** implement strict biosecurity, quarantine (14–21 days), and microbiological diagnosis (231). Reduce density and improve water quality. Adjust seasonal management at temperatures < 20 °C (231).

**Hypoproteinemia:** formulate diets with appropriate protein levels by production stage; evaluate digestibility and amino acid balance. Control hepatotoxins: aflatoxins < 20 ppb, peroxides < 5 meq/kg (234). Treat enteropathies and intestinal parasitosis. Avoid prolonged fasting; maintain regular feeding protocols.

##### 3.6.4.5 Cholesterol

Plasma cholesterol is essential for membrane structure, steroid hormone synthesis, bile acids, and fat-soluble vitamin metabolism (236, 237). In Tilapia it is particularly relevant when plant-based diets are used, which naturally contain little cholesterol. Plasma levels reflect the balance among intake, hepatic synthesis, metabolism, and biliary excretion (237).

**Operational range:** 100–200 mg/dl in healthy tilapia (204, 236, 238).

**EPI** **→**

**↑Hypercholesterolemia (>200 mg/dl):** hyperlipid diets (>10% lipids) or excessive dietary cholesterol (237); plant diets without bile acid supplementation (impaired emulsification/lipid metabolism) (237); hepatic dysfunction due to hepatotoxins (234); acute stress from air exposure altering endogenous synthesis (228); oxidized oils causing dyslipidemia (236).

**↓Hypocholesterolemia (< 100 mg/dl):** hypolipidemic plant extracts (236); prolonged fasting (>5 days) (235); severe hepatic dysfunction (234); intestinal malabsorption due to enteritis/parasitism (204); lipid-deficient diets (< 3%) (225).

**DOM** **→** **Feeding and nutrition:** formulations, cholesterol/lipid content, digestibility (237). **Economic and productive:** pressure to reduce costs by lowering protein/lipids. **Health and sanitary:** hepatopathies, intestinal parasitosis, enteropathies (234). **Natural resources (water):** heavy metals/water quality affecting the liver. **Animal:** acute stress due to air exposure (228). **Transport and logistics:** poor handling generating stress. **Human resources:** formulation, management, and capturing errors.

**Individual scoring system (Pi)**

**0 (normal):** 100–200 mg/dl.

**1 (mild alteration):** 80–100 or 200–230 mg/dl.

**2 (moderate–severe alteration):** < 80 or >230 mg/dl, or changes accompanied by clinical signs (lethargy, hepatomegaly, poor body condition).

**Population criterion**

If **≥20%** of the lot shows **Pi** **≥** **1**, there is population-level risk of hepatic dysfunction, nutritional errors, or acute stress. Any **Pi** **=** **2** requires immediate review of diet, hepatic health, and handling.

**Sentinel AWE criterion**

If **≥2 fish** show **Pi** **=** **2** in the same lot, this indicates an **AWE** linked to nutritional failures, hepatotoxins, or stressful handling. A progressive rising or falling pattern suggests systemic risk.

**Operational corrective action**

**Hypercholesterolemia:** adjust total lipids to 6%−8% (growth) or 4%−6% (maintenance). Supplement cholesterol (0.5%−1.0%) and bile acids (0.05%−0.1%) in plant-based diets (237). Improve ingredient quality: peroxides < 5 meq/kg, aflatoxins < 20 ppb (234). Reduce handling stress: air exposure < 3 min; eugenol 30–50 mg/L (228). If persistent, perform hepatic diagnostics.

**Hypocholesterolemia:** correct lipid deficiency (minimum ~6% as a practical target for many growth contexts); treat enteritis/parasitism and improve lipid digestibility; reduce fasting to a maximum of 24–48 h pre-harvest (235).

##### 3.6.4.6 Triglycerides

Triglycerides are the main form of lipid energy storage and transport. They are produced in the liver and circulate in VLDL and chylomicrons (236). Plasma levels reflect the balance among lipid intake, hepatic synthesis, mobilization from stores, and peripheral oxidation (235). They indicate energetic status, lipid metabolism, and nutritional/environmental metabolic stress (231).

**Operational range:** 100–300 mg/dl in healthy tilapia (204, 231). Juveniles may show higher values (200–400 mg/dl) (238).

**EPI** **→**

**↑Hypertriglyceridemia (>300 mg/dl in adults;** **>400 mg/dl in juveniles):** hyperlipid diets (>10% lipids) or hyperprotein diets (>40%) causing hepatic metabolic overload (225), chronic overfeeding; excessive lipid mobilization during winter thermal stress (231); fasting followed by abrupt refeeding (235); hepatic dysfunction altering VLDL metabolism (234); metabolic stress from poor water quality or excessive density.

**↓Hypotriglyceridemia (< 100 mg/dl):** prolonged fasting with depletion of reserves (235); lipid-deficient diets (< 3%) or insufficient energy (225); hypolipidemic plant extracts (236); severe hepatic dysfunction (234); chronic disease with negative energy balance (204); intestinal malabsorption (235).

**DOM** **→** **Feeding and nutrition:** diets exceeding hepatic metabolic capacity, chronic overfeeding, abrupt refeeding post-fasting, lipid/energy deficiency (204, 225, 235). **Human resources:** operational errors in feeding rate adjustment, temperature-based feeding, fasting/refeeding management (225, 235). **Natural resources (water):** winter thermal stress affecting appetite/metabolism and lipid mobilization (231). **Health and sanitary:** chronic disease/parasitism causing prolonged negative energy balance (204). **Therapeutics:** experimental/functional use of hypolipidemic plant extracts (236). **Animal:** density/handling increasing energetic demand and metabolic stress (204, 231). **Economic and productive:** cost-driven unbalanced feeds; prolonged pre-harvest fasting under economic pressure (204, 225, 235).

**Individual scoring system (Pi)**

**0 (normal):** 100–300 mg/dl.

**1 (mild alteration):** 80–100 or 300–350 mg/dl.

**2 (moderate–severe alteration):** < 80 or >350 mg/dl, or accompanied by clinical signs (lethargy, poor body condition, hepatomegaly).

**Population criterion**

If **≥20%** of the lot shows **Pi** **≥** **1**, this signals nutritional or hepatic failure risk. Any **Pi** **=** **2** requires immediate review of diet, feeding protocols, and hepatic health.

**Sentinel AWE criterion**

If **≥2 fish** show **Pi** **=** **2**, this indicates an **AWE** linked to nutritional errors (energy excess/deficit), thermal stress, hepatopathies, or poor water quality. A decreasing trend (< 100 mg/dl) or increasing trend (>300 mg/dl) signals systemic metabolic risk.

**Operational corrective action**

Adjust lipids to ~6%−8% and protein to ~28%−35% (225). Balance dietary energy:protein. Apply optimal feeding rates and adjust for temperature. Avoid overfeeding. Under winter thermal stress: smaller, more digestible rations and adjusted feeding frequency. Limit fasting and implement gradual refeeding.

##### 3.6.4.7 Albumin

Albumin is the most abundant plasma protein, synthesized exclusively in the liver, representing 40%−60% of total proteins (201, 229). It maintains oncotic pressure, transports lipids, hormones, and drugs, and exerts antioxidant functions (204). Its concentration reflects hepatic synthetic capacity, protein nutritional status, and renal/intestinal integrity. Albumin is one of the best markers of subclinical liver dysfunction and protein malnutrition in batches of tilapia.

**Operational range:** 1.5–3.5 g/dl in healthy adult tilapia (201, 204, 229).

**EPI** **→**

**↑Hyperalbuminemia (>3.5 g/dl):** dietary supplementation with immunostimulants (222). High-quality protein diets increasing hepatic synthesis (222).

**↓Hypoalbuminemia (< 1.5 g/dl):** protein-deficient diets (< 25% CP) or low digestibility (232, 233); hepatic dysfunction due to aflatoxins, oxidized oils, heavy metals (234); protein loss via nephropathies/enteropathies; intestinal malabsorption due to inflammation or parasitism.

**DOM** **→** **Feeding and nutrition:** protein deficiency/low digestibility (232, 233); additives improving hepatic synthesis (probiotics) (222). **Natural resources (water):** hepatotoxins in water, heavy metals (234), elevated CO_2_ causing nephropathies (206). **Health and sanitary:** hepatotoxicity from aflatoxins/oxidized oils (234); enteropathies from infection/parasitism (204). **Therapeutics:** inappropriate use of methyltestosterone (234); antibiotics/antiparasitics based on diagnosis (204). **Human resources:** errors in feed formulation, storage, or delivery (234). **Animal:** excessive densities (232). **Economic and productive:** cost-cutting compromising dietary protein (232).

**Individual scoring system (Pi)**

**0 (normal):** 1.5–3.5 g/dl.

**1 (mild alteration):** 1.3–1.5 or 3.5–3.8 g/dl.

**2 (moderate–severe alteration):** < 1.3 or >3.8 g/dl, or accompanied by compatible signs (edema, ascites, cachexia, poor body condition).

**Population criterion**

If **≥20%** show **Pi** **≥** **1**, this is a nutritional/hepatic or water/management alert.

If **≥10%** show **Pi** **=** **2**, systemic hepatic or nutritional failure is likely; review diets and water quality urgently.

**Sentinel AWE criterion**

If **≥2 fish** show **Pi** **=** **2**, this constitutes an **AWE**.

**Operational note**

A single fish with **Pi** **=** **2** warrants confirmation and immediate environmental/clinical review, even if it does not meet the AWE threshold.

**Operational corrective action**

**Hyperalbuminemia:** reduce densities and increase pond depth; improve water exchange and monitor evaporation (conductivity/salinity). Review dietary supplementation—continue only if intentional and beneficial (222). Maintain adequate dietary protein and balanced amino acids.

**Hypoalbuminemia:** reformulate diets with adequate protein and high digestibility; balance amino acids (lysine, methionine, and threonine). Prevent hepatotoxicity from aflatoxins and monitor heavy metals. Ensure appropriate therapeutic management for sex reversal and avoid methyltestosterone misuse. Treat enteropathies with antibiotics/antiparasitics as indicated by diagnosis.

##### 3.6.4.8 Globulins

Globulins include immunoglobulins, complement proteins, acute-phase proteins, transport proteins, and coagulation factors (204, 229). They represent 40%−60% of total proteins and reflect immune status, chronic infections, persistent inflammation, and antigenic exposure (231).

**Operational range:** 1.5–3.0 g/dl (15–30 g/L) in healthy adult tilapia (201, 204, 229).

**EPI** **→**

**↑** **Hyperglobulinemia (>3.0 g/dl):** chronic bacterial infections (Streptococcus, Aeromonas, Flavobacterium); winter stress with increased sanitary challenges (231); chronic parasitosis (protozoa, monogeneans, crustaceans) (204); chronic inflammation due to poor water quality (ammonia >1 mg/L, nitrite >0.5 mg/L, extreme pH) (204); immunostimulant supplementation (β-glucans, herbal extracts, probiotics) (222, 239).

**↓** **Hypoglobulinemia (< 1.5 g/dl):** immunosuppression due to severe chronic stress (extreme density, persistent hypoxia, chronic ammonia) (232); nutritional deficiencies (protein; vitamins A–C–E; Zn; Se) (140, 145); immunosuppressive viral diseases; environmental immunosuppressants (heavy metals, pesticides, mycotoxins) (204); prolonged fasting (>7 days) (235).

**DOM** **→** **Health and sanitary:** chronic infections/parasitism and persistent inflammation altering globulins depending on immune phase (204, 231). **Animal (population):** density, rough handling, introduction without quarantine, continuous stress modulating globulin production (204, 232). **Natural resources (water -environment domain-):** ammonia/nitrite, extreme pH, suspended solids, winter conditions modulating immunity and globulin variability (204, 231). **Feeding and nutrition:** deficient protein/vitamins/minerals depressing immunoglobulin production (204, 232). **Therapeutics:** immunostimulants that intentionally modulate globulins (222, 239).

**Individual scoring system (Pi)**

**0 (normal):** 1.5–3.0 g/dl.

**1 (mild–moderate alteration):** 1.2–1.5 or 3.0–3.5 g/dl.

**2 (moderate–severe alteration):** < 1.2 or >3.5 g/dl.

**Population criterion**

If **≥20%** show **Pi** **≥** **1**, this is a population alert requiring domain review.

If **≥10%** show **Pi** **=** **2**, risk of a population-level AWE is high.

**Sentinel AWE criterion**

If **≥2 fish** show **Pi** **=** **2**, this constitutes an **AWE**.

**Operational note**

A single fish with **Pi** **=** **2** strongly suggests an active chronic infection, severe immunosuppression, or a major environmental failure; initiate diagnostics and repeat the panel.

**Operational corrective action**

**Hyperglobulinemia:** strict biosecurity (quarantine, disinfection, vector control). Etiologic diagnosis (bacteriology, parasitology). Correct water quality (ammonia < 0.5 mg/L, nitrite < 0.2 mg/L, DO >5 mg/L). Reduce density. Targeted treatments (antibiotics per antibiogram; specific antiparasitics). Adjust immunostimulant supplementation to avoid hyperactivation.

**Hypoglobulinemia:** correct density and oxygenation (DO >5 mg/L). Restore appropriate nutrition (protein, vitamins, minerals by life stage). Remove environmental immunosuppressants (metals, pesticides, and mycotoxins). Strengthening biosecurity and viral prevention. Program physiological recovery with nutritional support.

##### 3.6.4.9 A/G ratio

The albumin/globulins ratio (A/G) is an integrated index reflecting the balance between hepatic albumin synthesis and immune globulin production (201, 204, 231). It is sensitive to hepatic function, protein nutrition, immune activation, and chronic inflammation. A/G shifts can identify liver–immune imbalance before absolute albumin or globulin values change (204).

**Operational range:** 0.8–1.5 in healthy tilapia (201, 204). A value ~1.0 indicates normal albumin–globulin equilibrium.

**EPI** **→**

**↑A/G (>1.5):** hypoglobulinemia due to chronic immunosuppression (severe stress, toxins, nutritional deficiencies) (232); optimal diets with high-quality protein and low antigenic load (222); “ultraclean” systems (UV/ozone) or stabilized biofloc reducing immune stimulation (204); post-infection recovery with still-low globulins.

**↓A/G (< 0.8):** hyperglobulinemia due to chronic bacterial/parasitic infections (231); chronic inflammation from poor water quality (204); winter stress increasing susceptibility (231); immunostimulant supplementation increasing globulins (222, 239); hypoalbuminemia due to hepatic dysfunction (234); protein-deficient diets with subclinical infections (231, 233).

**DOM** **→** **Health and sanitary:** chronic infections/parasitism and hepatopathies (aflatoxins, steatosis) driving A/G imbalance (231, 234). System hygiene, low antigenic load systems elevate A/G; biosecurity failures can reduce it (204, 222). **Feeding and nutrition:** protein or essential amino acid deficiencies reduce albumin and alter A/G; optimal nutrition stabilizes A/G (204, 233). **Natural resources (water -environment domain-):** ammonia/nitrite, extreme pH, hypoxia driving chronic inflammation and immune stress, shifting A/G (204, 232). **Animal (population):** extreme density and persistent stress; winter conditions altering adaptive immunity and hepatic synthesis (231, 232).

**Individual scoring system (Pi)**

**0 (normal):** 0.8–1.5.

**1 (mild alteration):** 0.7–0.79 or 1.5–1.8.

**2 (severe alteration):** < 0.7 or >1.8.

**Population criterion**

If **≥20%** show **Pi** **≥** **1**, this indicates a population-level immune or hepatic imbalance.

**Sentinel AWE criterion**

If **≥2 fish** show **Pi** **=** **2**, particularly with evidence of infection, impaired water quality, or hepatic/metabolic signs, this constitutes an **AWE**, regardless of lot size.

**Operational corrective action**

Immediate diagnostics (microbiology, parasitology, and hepatic biomarkers). Improve water quality (ammonia, nitrite, DO, pH). Optimize dietary protein and amino acid balance. Implement strict biosecurity. Apply targeted treatment as indicated (antibiotics per antibiogram; antiparasitics). Evaluate potential hepatotoxins (feed, water). In intensive systems, adjust temperature, density, and feeding rate.

##### 3.6.4.10 Plasma ammonia

Plasma ammonia reflects nitrogen excretion efficiency, gill function, and water quality. Teleosts excrete ammonia mainly via branchial diffusion; accumulation indicates elevated environmental exposure, gill dysfunction, hypoxia, or increased protein catabolism. Ammonia is neurotoxic, alters energy metabolism, and can interact with salinity by modulating antioxidant enzymes (224, 226).

In healthy tilapia, values ** < 100** **μmol/L** (< 0.17 mg/dl) are reported under good water quality (204).

**EPI** **→**

**↑Hyperammonemia (>200** **μmol/L):** exposure to high environmental ammonia due to excessive density, overfeeding, insufficient biofiltration, poor exchange, excessive organic loading; gill dysfunction from toxins or parasites reducing ammonia excretion (224). Hypoxia limiting branchial transport/excretion; excessive protein catabolism from hyperprotein diets, prolonged fasting, or catabolic disease (204). Ammonia–salinity interactions disrupting ion control/osmoregulation and antioxidant responses (224). Cyanobacterial blooms altering nitrogen metabolism (226).

**↓** **Hypoammonemia (< 50** **μmol/L):** positive indicator of good water quality, low density, and effective biofiltration; stable RAS nitrification (204). **Toxicological note:** 96 h LC50 for ammonia-N = 0.86 mg/L in Nile tilapia (224). Chronic exposures >0.5 mg/L induce oxidative stress and hepatic damage.

**DOM** **→** **Natural resources (water):** organic load, ammonia, exchange, biofiltration, salinity, and ammonia–salinity interactions (224, 226). **Animal:** density, sampling frequency/intensity, hypoxia exposure (204). **Feeding and nutrition:** hyperprotein diets, overfeeding, abrupt ration changes (204). **Health and sanitary:** diagnosis/treatment of gill parasitosis; prevention of toxin-related gill injury (224). **Therapeutics:** control of catabolic disease, support for gill failure (226). **Human resources:** training in organic load management, feeding rates, ammonia monitoring, and biofiltration control. **Economic and productive:** density pressure, insufficient investment in biofiltration and monitoring (224).

**Individual scoring system (Pi)**

**0 (normal):** plasma ammonia < 100 μmol/L with adequate environmental parameters and no compatible clinical signs.

**1 (mild alteration):** 100–200 μmol/L without evident clinical signs, or a single risk factor identified.

**2 (moderate–severe alteration):** ≥200 μmol/L, or elevated values accompanied by hypoxia, gill dysfunction, extreme density, algal blooms, ammonia–salinity interactions, or compatible clinical signs (gasping, lethargy, loss of equilibrium).

**Population criterion**

If **≥20%** of fish show **Pi** **≥** **1**, this indicates population-level deterioration of nitrogen metabolism and requires immediate intervention (exchange, biofiltration, density reduction).

**Sentinel AWE criterion**

If **≥2 fish** show **Pi** **=** **2** in the same sampling group, this constitutes an **AWE** associated with environmental/gill failure and mandates immediate corrective actions.

**Operational corrective action**

Maintain NH_3_-N < 0.5 mg/L by increasing exchange (10%−20%/day), optimizing biofiltration, and reducing organic load (224). Adjust stocking density to true system capacity and improve aeration to prevent hypoxia. Correct overfeeding and avoid hyperprotein diets when the system cannot process nitrogen loading. Treat gill parasitosis when indicated (formalin 25 mg/L for 1 h) and remove environmental toxins (224). Control ammonia–salinity interactions: ammonia < 0.5 mg/L and gradual salinity acclimation (224). Strengthen daily monitoring of ammonia, nitrite, DO, and salinity with staff training.

##### 3.6.4.11 Na^+^, Cl^−^, K^+^, and osmolality

Plasma osmolality and sodium, chloride, and potassium concentrations are essential for osmoregulation, neuromuscular function, and acid–base balance. They depend on integrated gill–kidney–intestine function and respond to salinity challenges, dehydration, and branchial or renal injury (206, 210). In freshwater tilapia, reference osmolality values of **270–330 mOsm/kg** have been described (206). For plasma electrolytes, approximate reference values include **Na**^**+**^ **~130–160 mmol/L**, **Cl**^**−**^ **~110–140 mmol/L**, and **K**^**+**^ **~3.5–6.0 mmol/L**, with variation related to water salinity and culture context (204, 210).

**EPI** **→**

**↑** **Hyperosmolality/hypernatremia:** exposure to elevated salinities without adequate acclimation; abrupt transfers to brackish/marine water; undetected saline intrusion at the intake; dehydration associated with excessive density and shallow systems with high evaporation; and body water loss due to toxin-induced gill dysfunction or poor water quality (206, 210, 211).

**↓** **Hypoosmolality/hyponatremia:** hemodilution secondary to repeated blood sampling or excessive sampling volumes; osmoregulatory failure due to gill injury (elevated ammonia and nitrite, parasitosis, infections); and use of very low-conductivity water without mineral supplementation (204, 206, 224).

**Ammonia–salinity interactions:** combined exposure to ammonia and elevated salinity disrupts ionic control, osmoregulation, and antioxidant responses, potentiating the physiological impact of each factor (224).

**DOM** **→** **Natural resources (water -environment domain-):** salinity/conductivity management, ammonia and nitrite control, prevention of saline intrusion, and intake water quality (204, 206, 210, 224). **Animal:** stocking density; frequency/intensity of blood sampling; handling during transfers between systems with different salinities (206, 211). **Health and sanitary:** diagnosis/control of gill injury due to toxins/pathogens compromising osmoregulation (206, 224). **Therapeutics:** sampling and treatment designs minimizing hemodilution and gill damage; specific support for osmoregulatory failure (206, 224). **Human resources:** staff training in gradual salinity acclimation, safe blood sampling limits, and routine monitoring of ionic and osmotic parameters (204, 206, 210). **Economic and productive:** production pressure sustaining densities above recommendations and limiting investment in salinity/osmolality monitoring (211).

**Individual scoring system (Pi)**

**0 (normal):** osmolality 270–330 mOsm/kg; Na^+^ 130–160 mmol/L; Cl^−^ 110–140 mmol/L; K^+^ 3.5–6.0 mmol/L, without compatible clinical signs of osmoregulatory injury.

**1 (mild alteration):** ~10%−20% deviation from the reference range in one parameter (osmolality or one electrolyte), without evident clinical signs and without concurrent abnormalities in other internal/external indicators.

**2 (moderate–severe alteration):** at least one parameter deviating by >20% from the reference range, or two or more electrolytes/osmolality out of range, and/or presence of compatible signs of osmoregulatory failure (marked edema, ascites, ventilatory abnormalities, or loss of equilibrium), interpreted jointly with other internal and external indicators.

**Population criterion**

If **>20%** of sampled fish show **Pi** **≥** **1** for this indicator, this is interpreted as population-level osmoregulatory deterioration requiring intervention in water management, density, and habitat quality.

**Sentinel AWE criterion**

If the sample contains **≥2 fish with Pi** **=** **2**, this constitutes an osmoregulatory **AWE**, regardless of pond/lot size, and immediate corrective measures must be activated.

**Operational corrective action**

Prioritize simultaneous correction of salinity, water quality, and handling. Implement gradual salinity acclimation during transfers or suspected saline intrusion, with continuous conductivity monitoring and verification of the water source. Adjust stocking density and effective pond depth to reduce dehydration and osmotic stress (e.g., depths >1.5 m and densities within system-specific recommended ranges). Correct water quality: maintain total ammonia < 0.5 mg/L and nitrite < 0.2 mg/L and prevent combined ammonia–salinity exposures that destabilize osmoregulation (206, 224). Limit blood sampling frequency and volume to ** < 1% of body weight per event**, and space sampling events (≥2 weeks), especially for high-value or low-stress lots (206). Train staff on acclimation protocols, salinity management, and sampling procedures, integrating this indicator into routine dashboards together with other internal parameters.

#### 3.6.5 Hepatic, muscle, and oxidative stress indicators

Hepatic and muscle enzymes (ALT, AST, and LDH) and oxidative stress biomarkers—including malondialdehyde (MDA), the antioxidant enzymes superoxide dismutase (SOD), catalase (CAT), glutathione peroxidase (GPx), and protein carbonyls—constitute an integrated set of indicators reflecting cellular metabolic function, tissue integrity, and redox balance in tilapia. Transaminases and LDH are intracellular enzymes released into plasma when hepatocellular or muscular injury occurs, or when systemic cytotoxicity is present, making them sensitive sentinels of environmental, toxicological, or metabolic insults. In parallel, MDA and protein carbonyls quantify the degree of lipid peroxidation and irreversible protein oxidation, respectively, providing a direct measure of cumulative oxidative damage. Finally, SOD, CAT, and GPx represent the defensive response against reactive oxygen species (ROS), showing compensatory increases in early stages of stress or decreases due to enzymatic exhaustion under advanced or prolonged challenges. Collectively, these parameters allow high-resolution characterization of the magnitude and nature of physiological stress, support identification of risk factors across EPI-DOM management domains and enable evidence-based operational decisions to prevent or resolve adverse welfare events.

##### 3.6.5.1 ALT/AST/LDH (hepatic and muscle enzymes)

Transaminases (AST, ALT) and lactate dehydrogenase (LDH) are intracellular enzymes released into plasma during hepatocellular injury, muscle damage, or generalized cytotoxicity. They are sensitive biomarkers of hepatic injury, oxidative stress, hypoxia, and xenobiotic toxicity (218, 226, 240). Elevation of these enzymes indicates cellular leakage and loss of tissue integrity.

**Operational range:**

**AST:** 20–100 U/L

**ALT:** 5–40 U/L

**LDH:** 50–300 U/L in healthy tilapia (201, 204).

Values vary with temperature, metabolic activity, and nutritional status.

**EPI** **→**

**↑** **Elevation:** ALT and AST increase with exposure to cyanobacterial toxins—microcystin-driven hepatotoxicity (226); exposure to cigarette-butt leachates—nicotine and organic toxins (218); diets containing oxidized fish oil—ALT and AST increases (240); methylmercury exposure—tissue injury and elevated hepatic enzymes (207). General cytotoxicity with LDH increase under toxicant exposure (218); lipid peroxidation associated with oxidative stress (217); chronic hypoxia—hepatocellular and muscle injury (204); viral infections with hepatocellular necrosis (TiLV or others) (204); muscle injury due to rough handling, impacts, or air exposure (219).

Drivers: hepatotoxicity from toxins, lipid peroxidation, hypoxia, oxidative stress, viral infections (hepatocellular necrosis), oxidized lipids in diets, and handling-related muscle injury.

**DOM** **→** **Natural resources (water):** toxic contaminants (microcystins, heavy metals) and poor water quality promote hepatotoxicity (207, 226). **Feeding and nutrition:** use of oxidized or improperly stored ingredients elevates transaminases (240). **Health and sanitary:** viral diseases and systemic cytotoxicity increase AST/ALT/LDH (204, 218). **Animal:** rough handling causes muscle damage with LDH elevation (219). **Human resources:** operational errors, poor feed storage, water contamination, or traumatic capture contribute to hepatic and muscle injury (218, 240). **Economic and productive:** cost-cutting practices associated with low-quality inputs increase hepatotoxicity risk (240).

**Individual scoring system (Pi)**

**0 (normal):** values within the physiological range or ≤ 1.0 × the reference value.

**1 (mild–moderate alteration):** >1.0 × and < 2.0 × the reference value. Consistent with adaptive responses, early physiological stress, or an AWE present without immediate life-threatening compromise.

**2 (severe alteration):** ≥2.0 × the reference value. Indicates tissue damage or functional compromise with risk of substantial pain, suffering, or mortality.

**Population criterion**

**Alert:** >25% of individuals with **Pi** **=** **1**.

**Critical:** ≥10% of the lot with **Pi** **=** **2**, or any sustained upward trend across two consecutive sampling events.

**Sentinel AWE criterion**

If **one fish** in the sample shows **Pi** **=** **2**, this constitutes an **AWE**. Concurrent elevations of **AST** **+** **ALT** **+** **LDH** suggest severe hepatotoxicity or imminent organ failure.

**Operational corrective action**

Immediately remove the most likely hepatotoxic source (microcystins, leachates, heavy metals). Assess feed quality: measure peroxide value, discard oxidized lots, and improve storage. Implement targeted antioxidant supplementation (vitamin E, vitamin C, and selenium). Increase oxygenation (DO >5 mg/L) and reduce stocking density (204). Apply strict biosecurity and perform viral diagnostics if infection is suspected. Train personnel in gentle handling and non-traumatic capture procedures.

##### 3.6.5.2 Malondialdehyde (MDA)

MDA is an end product of lipid peroxidation and functions as a direct biomarker of oxidative damage to cellular membranes. MDA formation occurs when reactive oxygen species attack polyunsaturated fatty acids, generating toxic byproducts that reflect systemic oxidative stress (217, 218). Elevated MDA indicates membrane integrity disruption, inflammation, and increased free radical burden.

**Operational range:**

** < 2.0 nmol/mg protein**, or

** < 5.0** **μmol/L in plasma**, under optimal culture conditions (210, 217).

**EPI** **→**

**↑MDA (>3.0 nmol/mg protein):** toxicity from mercuric chloride (217); exposure to cigarette-butt leachates (218); heavy metals such as cadmium and lead (217); chronic hypoxia increasing ROS generation (204); thermal stress (204); antioxidant-deficient diets (217); excessive UV radiation (204); high stocking densities (211); oxidized oils in diets (225, 240).

**DOM** **→** **Natural resources (water):** heavy metals or toxic compounds that induce lipid peroxidation (217, 218). **Natural resources (water -environment domain-):** hypoxia, UV exposure, and thermal fluctuations increasing oxidative stress (204). **Feeding and nutrition:** antioxidant-deficient diets or oxidized oils promoting peroxidation (217, 225, 240). **Animal:** high densities increasing physiological stress and ROS production (211). **Human resources:** failures in feed storage, biosecurity, or contaminant surveillance (218, 240). **Economic and productive:** cost-saving that reduces antioxidant supplementation or enables use of oxidized inputs (225).

**Individual scoring system (Pi)**

**0 (normal):** values within the physiological range or ≤ 1.0 × the reference value.

**1 (mild–moderate alteration):** >1.0 × and < 2.0 × the reference value; consistent with adaptive responses, early physiological stress, or an AWE present without immediate life-threatening compromise.

**2 (severe alteration):** ≥2.0 × the reference value; indicates severe oxidative damage to lipid membranes with high functional risk, associated with physiological suffering and potential survival impact if not immediately addressed.

**Population criterion**

**Alert:** >25% of the lot with **Pi** **=** **1**.

**Critical:** ≥10% with **Pi** **=** **2**, or sustained upward trend across two consecutive sampling events.

**Sentinel AWE criterion**

If **one fish** in the sample shows **Pi** **=** **2**, this constitutes an **AWE**. Elevated MDA should be interpreted alongside other oxidative stress markers (SOD, CAT, GPx) and/or hepatic injury markers.

**Operational corrective action**

Eliminate the source of heavy metal contamination or toxic leachates. Improve water quality and prevent hypoxia; maintain DO >5 mg/L. Supplement antioxidants: vitamin E (100–200 mg/kg), vitamin C (200–500 mg/kg), selenium (0.3–0.5 mg/kg) (217). Use fresh oils and store feeds under appropriate conditions. Reduce stocking density, provide pond shading, and manage temperature. Train staff on contaminant prevention and input rotation.

##### 3.6.5.3 Superoxide dismutase (SOD)/Catalase (CAT)/Glutathione peroxidase (GPx)

The antioxidant enzymes SOD, CAT, and GPx constitute the primary enzymatic defense against reactive oxygen species (ROS). SOD converts superoxide (${\text{O}}_{2}^{-}$) into H_2_O_2_, while CAT and GPx degrade H_2_O_2_ to water and oxygen, preserving cellular redox homeostasis (210, 241). Shifts in activity reflect oxidative stress, protective adaptive responses, or enzymatic exhaustion under prolonged challenge or tissue injury.

**Operational range:** in liver of healthy tilapia, values depend on tissue, diet, age, and environmental conditions (217, 241):

**SOD:** 50–150 U/mg protein

**CAT:** 30–100 U/mg protein

**GPx:** 20–80 U/mg protein.

**EPI** **→**

**↑** **Activity (protective induction):** exposure to cigarette-butt leachates (compensatory response) (218); antioxidant supplementation (β-carotene, *Phyllanthus emblica, Nigella sativa*, genistein, BergaPur^®^) (217, 222, 241); adaptive response to moderate oxidative stress.

**↓** **Activity (enzymatic exhaustion):** decreased SOD and CAT under salinity stress (210); depletion due to severe chronic oxidative stress. Ammonia–salinity interactions alter hepatic enzymes (224). Deficiency of cofactors Cu, Zn, Mn, Se (204).

**DOM** **→** **Natural resources (water):** xenobiotic contamination, salinity shifts, and ammonia affecting enzymatic activity (210, 218, 224). **Health and sanitary:** environmental toxicants triggering oxidative stress or depleting defenses (218). **Feeding and nutrition:** diets deficient in antioxidants or essential mineral cofactors (204, 217). **Animal:** high density or other conditions increasing ROS and exhausting antioxidant capacity (210). **Economic and productive:** cost reduction that removes adequate antioxidant supplementation (222).

**Individual scoring system (Pi)**

**0 (normal):** values within the physiological range or ≤ 1.0 × the reference value.

**1 (mild–moderate alteration):** activity increased or decreased with deviation ≤ 1.8 × relative to reference.

**2 (severe alteration):** increase or decrease ≥2.0 × relative to reference; indicative of marked oxidative stress or enzymatic exhaustion.

**Population criterion**

**Alert:** >25% of the lot with **Pi** **=** **1**.

**Critical:** ≥10% of the lot with **Pi** **=** **2**, or a sustained alteration pattern across two consecutive sampling events.

**Sentinel AWE criterion**

Any individual shows **Pi** **=** **2**, or SOD–CAT–GPx display a pattern of initial elevation followed by a marked decline, consistent with exhaustion.

**Operational corrective action**

Eliminate contamination sources (xenobiotics, leachates, metals). Adjust salinity and ammonia to physiological levels; improve aeration. Supplement antioxidants: vitamin E (200–400 mg/kg), vitamin C (400–600 mg/kg), selenium (0.3–0.5 mg/kg) (217). Include mineral cofactors: Cu, Zn, Mn, and Se. Reduce stocking density and minimize concurrent stressors.

##### 3.6.5.4 Protein carbonyls (carbonylated proteins)

Protein carbonyls are products of irreversible protein oxidation induced by reactive oxygen species (ROS). Carbonylation alters the function and stability of structural and enzymatic proteins and is a robust marker of chronic oxidative damage (210, 218). Increased levels indicate sustained oxidative stress, cellular aging, and accumulation of molecular injury.

**Operational range:** < 1.5 nmol/mg protein in healthy tilapia (210). Values vary with age, antioxidant diet, and environmental exposure.

**EPI** **→**

**↑Protein carbonyls (>2.5 nmol/mg protein):** increased with exposure to heavy metals such as mercury and cadmium (207, 217); increased with xenobiotics (pesticides, industrial leachates) (218); elevated under chronic oxidative stress due to poor water quality, antioxidant-deficient diets, high stocking densities, thermal stress, or hypoxia (210). Linked to aging (204). Associated with oxidized lipids in diets (240).

**DOM** **→** **Genetics and reproduction:** broodstock aging and cumulative oxidative damage (204). **Natural resources (water):** heavy metals, xenobiotics, and conditions promoting oxidative stress (hypoxia, thermal fluctuations) (207, 218). **Health and sanitary:** environmental toxicants producing irreversible protein oxidation (218). **Feeding and nutrition:** oxidized lipids and/or antioxidant deficiency (217, 240). **Animal:** excessive density, chronic stress, unmitigated UV exposure (204, 211). **Economic and productive:** low-quality ingredients and practices that increase chronic stress (240).

**Individual scoring system (Pi)**

**0 (normal):** values within physiological range (< 1.5 nmol/mg protein).

**1 (mild–moderate increase):** 1.5–2.5 nmol/mg protein.

**2 (severe increase):** >2.5 nmol/mg protein, indicative of irreversible oxidative damage.

**Population criterion**

**Alert:** >25% of the lot with **Pi** **=** **1**.

**Critical:** ≥10% of the lot with **Pi** **=** **2**, or a persistent upward trend across two consecutive sampling events.

**Sentinel AWE criterion**

Any fish shows **Pi** **=** **2**, or MDA and protein carbonyls rise simultaneously, indicating systemic oxidative damage.

**Operational corrective action**

Eliminate heavy metal and xenobiotic sources; implement dynamic monitoring. Improve water quality; reduce density; correct hypoxia. Supplement antioxidants: vitamin E, β-carotene, vitamin C, and glutathione. Avoid oxidized oils; use fresh ingredients and ensure proper feed storage. Replace aged broodstock and improve their antioxidant nutrition. Provide partial shading and increase depth to reduce UV exposure.

**NOTE:** In the available tilapia literature, increases of approximately 1.2–1.8 × relative to enzyme reference values are consistently reported under moderate stress scenarios, sublethal exposure, or early compensatory responses. In contrast, increases ≥2 × are repeatedly associated with tissue injury, severe oxidative stress, or functional compromise. Therefore, this guide adopts ≥2 × as an operational threshold to classify severe alterations (Pi = 2), whereas smaller deviations are integrated under Pi = 1 and interpreted jointly with other welfare indicators.

## 4 Discussion

### 4.1 External indicators as early signals of risk and welfare disruption

External indicators constitute the first-tier detection system for welfare alterations in tilapia because they provide a visible, operationally accessible readout of the interaction between the fish and its production environment—the “conditions in which it lives and dies.” Skin lesions, scale erosion, gill alterations, changes in coloration, poor body condition, and morphological abnormalities should not be interpreted as isolated findings, but as phenotypic expressions of failures in one or more management categories. Within the EPI-DOM framework, their value lies in their capacity to integrate multiple risk factors—nutritional, environmental, sanitary, and handling-related—into an observable manifestation that can be monitored routinely and non-invasively. Operationally, external indicators function as field-based biological sensors: they often emerge before deep physiological injury becomes established, particularly under low-intensity chronic stress. In such scenarios, fish may maintain an apparent internal physiological stability through compensatory mechanisms while external damage is already evident. This early dissociation underscores the preventive value of external indicators and justifies their role as a structural component of the guide, especially for field-level decision-making and prioritization of corrective actions.

### 4.2 Internal indicators and physiological suffering: from adaptation to functional injury

Internal indicators deepen welfare assessment by revealing the organism's true physiological state beyond what is externally observable. Unlike external indicators, which signal that a problem exists, internal indicators allow characterization of its magnitude, duration, and functional consequences, positioning the fish along a continuum from physiological adaptation to chronic stress and irreversible damage. In early phases, many deviations reflect adaptive responses aimed at maintaining homeostasis under predictable production stressors. Moderate changes in glucose, lactate, cortisol, or antioxidant activity represent metabolic and endocrine adjustments that, if transient and reversible, do not necessarily imply severe welfare compromise. However, when alterations persist, intensify, or appear as a coherent multi-system pattern, physiology shifts from adaptation to physiological suffering. Operationally, physiological suffering is defined as a state in which compensatory mechanisms are no longer sufficient to preserve normal tissue function, resulting in cellular injury, persistent inflammation, profound metabolic disruption, and ultimately reduced survival and productive performance. Marked elevations of hepatic and muscle enzymes, sustained increases in oxidative stress biomarkers (e.g., MDA, protein carbonyls), and severe imbalances in plasma proteins provide objective evidence that this threshold has been exceeded. From an EPI-DOM perspective, physiological suffering is not spontaneous or inevitable; it represents the cumulative outcome of uncorrected failures across management categories and their cross-relationships with the Environment and Interaction domains.

### 4.3 Functional convergence between external and internal indicators

A central contribution of this guide is to demonstrate that external and internal indicators are not redundant, but complementary. External indicators enable early, operational detection, whereas internal indicators confirm, explain, and prioritize the physiological impact of the detected alterations. This convergence is particularly evident in scenarios involving inadequate handling, poor water quality, or nutritional errors, where external lesions co-occur with metabolic, immune, and oxidative disturbances. For instance, recurrent cutaneous erosion combined with declining body condition may coincide internally with hypoproteinemia, hypoalbuminemia, and altered A/G ratio—supporting that the problem is not merely mechanical but nutritional and hepatic in nature. Likewise, visible gill alterations often align with hyperlactatemia, plasma electrolyte shifts, and enzymatic patterns consistent with hypoxia and tissue stress. Joint interpretation reduces reliance on a single measurement type and strengthens diagnostic robustness by linking what is visible in the field to what is occurring physiologically.

### 4.4 Management implications: welfare as a reflection of the production system

Across indicator categories, a consistent pattern emerges: welfare alterations in tilapia are primarily associated with management decisions rather than inherent species limitations, because the main risk pathways documented for external damage, immune activation, metabolic disruption, and oxidative injury are directly shaped by routine operational choices (20, 211, 219). Feeding and nutritional management functions as a central axis due to its transversal effects on body condition and on internal patterns linked to protein status, lipid metabolism, and hepatic function—particularly under cost-driven formulation instability, inadequate protein/energy balance, oxidized inputs, or mycotoxin exposure—which consistently translate into coherent biochemical and enzymatic shifts (225, 232, 234–236). Health and sanitary management (biosecurity) shapes the system's antigenic load and the probability of persistent or mixed infections, which is reflected both in external lesions and hemorrhagic patterns and in internal immune profiles consistent with chronic inflammation or immunosuppression, reinforcing that etiologic control and preventive programs are integral to welfare stabilization (83, 96, 97, 180). Natural resource management—particularly water quality—acts as a constant welfare modulator by driving respiratory compensation and exhaustion dynamics under hypoxia and by impairing gill function under ammonia/nitrite accumulation, with downstream metabolic and ionic consequences that may occur without immediate mortality (191, 194, 196, 224). Animal (individual and population) management—including stocking density, capture/handling intensity, fasting/refeeding routines, and transport logistics—operates as a physiological stress amplifier that can convert reversible adaptation into functional injury, as evidenced by coordinated changes in ventilatory patterns, stress biomarkers, and immune ratios under repeated disturbance (43, 134, 137, 189, 219). Operationally, this systems-based interpretation improves causal traceability: for example, when recurring external erosions co-occur with tachypnea/gasping and rising lactate, the most parsimonious driver set typically centers on water-quality failures (oxygenation and nitrogenous load) plus density/handling stressors, prioritizing immediate aeration/exchange and biomass redistribution (189, 191, 196, 211). Likewise, when poor body condition and tegument compromise align with hypoproteinemia/hypoalbuminemia and enzyme/oxidative alterations, the pattern is more consistent with nutritional instability and hepatotoxic exposure than with purely mechanical trauma, prioritizing diet reformulation and feed-quality control alongside targeted diagnostics when indicated (217, 232, 234, 240). Finally, economic and productive decisions traverse all categories by influencing input quality, monitoring investment, and biomass pressure, making welfare an emergent property of the production system and its decision rules—not a trait of the fish—while the EPI-DOM guide provides a structured mechanism to identify intervention points, prevent progression from adaptation to physiological suffering, and reduce adverse welfare events (AWE) (20, 211, 219).

## 5 Practical implications for tilapia welfare management

### 5.1 From diagnosis to management: welfare as a decision tool

A major contribution of this guide is transforming welfare assessment into an operational decision tool rather than an exclusively diagnostic exercise. Integrating external and internal indicators enables a shift from a reactive logic—based on mortality or overt disease—to a preventive logic in which early deviations guide immediate adjustments in the production system. Welfare is thus treated as a functional gradient rather than a dichotomous state, where timely detection of mild deviations (Pi = 1) constitutes a critical intervention window to prevent progression toward physiological suffering (Pi = 2) and AWE. Effective implementation requires that monitoring is paired with clear decision rules (stop-criteria, escalation thresholds, and corrective action pathways), and that staff training emphasizes the physiological consequences of daily management decisions—not only technical procedures.

### 5.2 Operational priorities by management categories (EPI-DOM)

Integrated indicator analysis shows that interventions should be prioritized strategically because management categories do not contribute equally to welfare deterioration:

- Feeding and nutritional: a high-impact transversal driver. Errors in feed level, quality, or stability are consistently reflected in external indicators (body condition, skin integrity) and internal indicators (plasma proteins, lipid metabolism, hepatic enzymes). Nutritional correction often represents the first line of action when early welfare deterioration is detected.
- Health and sanitation (biosecurity): critical when patterns indicate persistent immune activation or chronic inflammation. In these cases, correcting diet or water quality alone is insufficient without etiological diagnosis, pathogen control, and reduction of antigenic pressure.
- Natural resource (Environment domain): water quality is a constant welfare modulator. Subclinical changes in dissolved oxygen, ammonia, or nitrite may not cause immediate mortality but can sustain chronic stress, oxidative damage, and hepatic dysfunction; therefore, parameters should be interpreted not only against toxicity thresholds but also through their cumulative physiological impacts.
- Animal (individual and population): strongly linked to the transition from adaptive stress to physiological suffering. Excessive densities, rough handling, prolonged fasting, and inadequate capture/transport amplify other stressors; small routine changes can yield meaningful physiological improvements.
- Economics and productivity: crosscuts all categories. Decisions focused exclusively on cost reduction or biomass maximization tend to translate, over time, into increased physiological stress and indirect productivity losses. The guide makes this trade-off visible and supports welfare-centered sustainability decisions.

### 5.3 Operational interpretation of physiological suffering

Explicit inclusion of physiological suffering establishes an ethical and technical threshold for immediate action. When internal indicators demonstrate functional injury—enzymatic, metabolic, or oxidative—it is no longer acceptable to postpone intervention under the argument that mortality or obvious clinical signs are absent. Physiological suffering is therefore treated as an operational criterion that compels urgent review of the management categories involved. By providing objective thresholds (Pi = 0, 1, 2) and linking them to corrective actions, the guide reduces subjectivity in decision-making and aligns welfare practice with internationally recognized welfare principles.

### 5.4 Using the guide across production contexts

The guide is deliberately modular to support application in laboratory, semi-intensive, intensive, and RAS/BFT systems without imposing an excessive technical burden. Not all indicators must be measured at all times; selection can be tailored to the predominant risk profile and operational capacity of each system. In laboratory contexts, internal indicators enable high-resolution validation of welfare impacts of procedures and treatments. In production settings, external indicators and a strategic subset of internal indicators can operate as a monitoring panel that escalates in complexity when risk rises.

### 5.4.1 Practical implications across geographical regions

Although the EPI-DOM logic is universal, implementation priorities vary across regions due to climate, production typology, diagnostic capacity, and regulatory/resource constraints:

- Climate and seasonality (tropical vs. temperate): temperature-driven shifts in dissolved oxygen dynamics, metabolic demand, and seasonal stress patterns alter the risk profile and the timing of monitoring intensity.
- Production typology (pond/semi-intensive vs. intensive/RAS/BFT): in pond systems, external indicators and water-quality-linked sentinels often provide the most feasible early-warning layer; in intensive and recirculating systems, continuous monitoring and targeted internal panels can be integrated more routinely.
- Diagnostic capacity (high vs. limited infrastructure): regions with limited laboratory access may prioritize a minimum viable panel (external indicators + respiratory rate + a small biochemical set), escalating to extended panels during suspect AWE scenarios; regions with stronger infrastructure can apply broader internal profiling for prevention and validation.
- Regulatory and market constraints: access to therapeutics, acceptable treatment options, and certification demands shape the feasible corrective-action pathways; therefore, the guide's emphasis on prevention and early intervention becomes especially valuable where pharmacological options are constrained.
- Resource constraints (energy, inputs, trained personnel): where backup aeration, continuous sensing, or frequent testing is limited, staff training and robust external-indicator surveillance become the primary leverage points, with internal testing used strategically to confirm and prioritize interventions.

This adaptability supports regional implementation without diluting scientific rigor: the framework standardizes interpretation logic while allowing context-specific selection of indicators and feasible corrective actions.

### 5.5 Implications for training and decision-making

Implementation requires a cultural shift in how welfare is conceptualized in aquaculture. By explicitly linking indicators to management categories and corrective actions, the guide strengthens evidence-based decision-making and reduces dependence on late or purely empirical interventions. In this sense, welfare becomes a system performance indicator comparable in relevance to classic production metrics.

## 6 Key message

**Tilapia's welfare does not fail in the fish: it fails in management**. Integrated evaluation of external and internal indicators allows early detection of when a production system is shifting from physiological adaptation to physiological suffering. The EPI-DOM framework transforms welfare into an operational management tool oriented toward preventing adverse events, optimizing decision-making, and aligning productivity with an ethical, scientifically grounded aquaculture.

## 7 Conclusions

This guide demonstrates that welfare assessment in Tilapia can and should move beyond approaches based solely on mortality, clinical disease, or isolated indicators. Systematic integration of external and internal indicators within the EPI-DOM framework enables early identification of management failures that, if uncorrected, progress from adaptive physiological responses to functional injury and physiological suffering. Biochemical, enzymatic, and oxidative indicators are not redundant relative to external indicators; they validate the physiological impact of handling, nutrition, environment, and production pressure, even in the absence of overt clinical signs. Adoption of clear operational thresholds (Pi = 0, 1, 2) distinguishes adaptation, reversible stress, and functional injury, providing an objective basis for timely intervention. This approach shifts responsibility from the animal to the production system—and thus to human decision-making—facilitating targeted, ethically justified corrective interventions.

## Data Availability

The original contributions presented in the study are included in the article/supplementary material, further inquiries can be directed to the corresponding author.
